# Supplementary material for: Comparative genome anatomy reveals evolutionary insights into a unique amphitriploid fish
Source: Nat Ecol Evol. 2022 Jul 11;6(9):1354–66. doi: 10.1038/s41559-022-01813-z (PMC9439954; doi:10.1038/s41559-022-01813-z)
Supplement: Supplementary file 1 — Supplementary Notes 1–7, Supplementary Figs. 1–21 and Supplementary Tables 1–23. [file 41559_2022_1813_MOESM1_ESM.pdf]

nature portfolio

https://doi.org/10.1038/s41559-022-01813-z

Supplementary information

Comparative genome anatomy reveals evolutionary insights into a unique amphitriploid fish

In the format provided by the authors and unedited

Supplementary Information

Supplementary Notes

Supplementary Note 1 | Polyploidy and genome size evaluation

The haplotype structures were extracted from heterozygous *k*-mer pairs using the Smudgeplots pipeline[1]. First, we produced *k*-mer frequency file using KMC[2] with *k*=21 from trimmed reads. Then, we searched for all heterozygous pairs of *k*-mers that differ at exact one nucleotide through a systematic scan of all input *k*-mers. To avoid sequencing errors of genomic *k*-mers, we filtered the *k*-mers with depth less than the depth of first trough in the *k*-mer frequency curve (10 for *C. gibelio* and 12 for *C. auratus*). Finally, we fed the heterozygous *k*-mer coverage file to the R script of the pipeline and plotted the smudgeplot and estimated ploidy. This tool performed gymnastics with the heterozygous *k*-mer pairs by comparing the sum of *k*-mer pair coverages (CovA + CovB) to their relative coverage (CovA / (CovA + CovB)). After that, we analyzed the two genomes with duplications and ploidy levels.

The genome size was estimated by using a *k*-mer-based method with jellyfish v2.3.0[3]. The Illumina short reads for each species were first used to count *k*-mers using jellyfish with different *k*-mer sizes (17, 19, 21, 23, and 25). The k-mers distributions were plotted and Genome size was estimated by:

$$\text{Genome Size} = k_{num}/k_{depth}$$

Where k_{num} is the number of *k*-mer, and k_{depth} is the expected depth of *k*–mer. It should be noted that we have also tried different softwares to estimate the genome size, including GCE[4], GenomeScope[1] and SOAPec[5], but the estimated sizes from different

methods and parameters varied greatly (such as 0.66, 1.3, 1.6 and 4.8Gb). Perhaps this could be attributed to that these softwares are still deficient in dealing with complex polyploids, such as misjudging the peak of the k-mer distribution. Therefore, we combined various information, such as polyploidy, flow cytometry results, and other published genome assembly results, to determine which peak corresponds to the haplotype size of the genome in the *k*-mer distribution and make an estimate of the genome size.

Supplementary Note 2 | Assembly quality evaluation

We used two approaches to evaluate the quality of the genome assemblies. Firstly, we mapped Illumina short reads to the genomes using BWA (v0.7.17-r1198-dirty)[6] with default parameters. The coverage ratio was estimated by SAMtools (v1.12)[7] and the mapping ratio was estimated by BEDTools[8]. Secondly, we performed BUSCO assessments for the genome sequences and the predicted gene models using BUSCO v5.2.2[9] with the actinopterygii_odb10 data set.

We also performed comparisons between the available *C. auratus* assemblies (Supplementary Table 23). First, the mapping rate of genomic reads was 99.85% and the mapping rate of RNA-seq reads ranged from 85.6% to 89.9% in our *C. auratus* assembly. These are comparable to 98.23% and 62.1%–90.4% in the genome version published in BMC Genomics (GCA_013115835.1), which has a genome size of 2.198 Gb. Second, we examined the extent of redundancy in different genome assemblies using the software purge_dups, including in our genome assembly (1.522 Gb),

GCA_014332655.1 (1.740 Gb) using nanopore long reads[10], and GCA_013115835.1 (2.198 Gb)[11]. The results showed that both previously published genomes had more obvious heterozygosity peaks than our assembly (Supplementary Fig. 20). This indicates more redundant assembly in heterozygous regions, which would inflate the assembled size. We then purged redundancy and extracted the haploid sequences from the two previous assemblies using purge_dups and obtained similar genome sizes (1.528 Gb for GCA_014332655.1 and 1.407 for GCA_013115835.1) (Supplementary Table 23). Therefore, these results indicate that the larger genome size in previous assemblies was mainly caused by redundancy in haploid sequences.

Supplementary Note 3 | Bias of subgenomes

Based on the CDS alignments obtained from the "Phylogenetic analysis of *C. gibelio* and *C. auratus*" step, we further removed those alignments with less than 150 bp informative sites and used the remaining ones for subsequent analysis. The ratio of nonsynonymous to synonymous substitutions (*Ka*/*Ks*) for each branch was estimated using the free ratio model of Codeml in PAML v4.9h[12] under default parameters. The entire analysis was performed using the species tree obtained from the previous analysis as a guide-tree.

Clean RNA-Seq reads were mapped to *C. gibelio* and *C. auratus* assemblies using HISAT2[13]. The expression levels were calculated using unique mapping reads and normalized using transcripts per million (TPM). We compared the expression of homoeologous genes based on transcripts TPM estimating by StringTie[14]. For each

homoeologue pair in each tissue and developmental stage, the A/B expression ratio was calculated and log transformed according to ($\log_{10}$((TPM.A+0.1)/(TPM.B+0.1))). Additionally, to investigate the effect of genome duplication on the expression of genes between *C. gibelio* and *C. auratus*, the expression ratio of homologous gene pairs between the two species was also assessed by ($\log_{10}$((TPM.*Cg*+0.1)/(TPM.*Ca*+0.1))).

Supplementary Note 4 | Genomic variations in the common ancestor of *Carassius*

We identified genes putatively lost in the *Carassius* species using a method described by Wang *et al.*[15]. Paired-end reads of the 10 *C. auratus*, six *C. gibelio*, and four *Sinocyclocheilus* were mapped to the *C. carpio* genome sequence (GCA_000951615.2) using BWA (Version 0.7.12-r1039)[6]. Paired-end reads of four *Sinocyclocheilus* were artificial FASTQs that derived from four genomes, *S. grahami* (one from ftp.cngb.org/pub/CNSA/data3/CNP0001478/CNS0353845/CNA0019203/ and the other was GCF_001515605.1), *S. rhinocerous* (GCF_001515625.1), and *S. anshuiensis* (GCF_001515605.1), by ArtificialFastqGenerator v1.0.0[16] using default settings. The gene was identified as loss in *Carassius* only if reads of all *Carassius* individuals could not be mapped to the *C. carpio* genome but corresponding reads from other species could be mapped. The genes that were detected to be specifically lost in *Carassius* were further manually checked by: (1) inspecting the read depth across the above individuals, and (2) examining the gene annotation in corresponding genome sequences. Moreover, to check whether the lost genes identified by our study

are present or absent in other four published assemblies, we applied a three-step test for each gene. Firstly, the amino acid (aa) sequences of the gene from *C. carpio* genome (GCA_018340385.1) were aligned to six *Carassius* genomes' sequences using tblastn (v2.10.1) to obtain the top best hits (identity > 50%), and *Danio rerio* and *Sinocyclocheilus graham* using diamond (v2.0.11.149, parameters: --sensitive --evalue 1e-10). Secondly, the gene structure and aa sequences of the targeted regions were extracted using annotated gff or genewise (v2.2.0). Thirdly, all the aa sequences were aligned together using mafft (v7.471), and a maximum likelihood tree was constructed using RAxML (v8.2.12). Then, the alignment sequences were checked with MEGA (v7.0.26) and the ML tree was checked with figtree (v1.4.4) to determine whether this gene was present in each genome assembly (Supplementary Fig. S21). The gene alignments and trees are available at figshare database (https://doi.org/10.6084/m9.figshare.19674843.v1).

These genes might be related to derived characteristics of *C. gibelio* and *C. auratus* (Supplementary Table 15). For example, one copy of cyclin-dependent kinase 2 (*cdk2*), also known as cell division protein kinase 2, is among the lost genes (Supplementary Fig. 10). This gene encodes the catalytic subunit of the cyclin-dependent kinase complex that is expressed in the G1-S phase of the cell cycle. A previous study found that *cdk2* knockout mice undergo fewer cell divisions and their germ cells arrest in the prophase of meiosis I[17].

Supplementary Note 5 | New non-coding elements in *C. gibelio*

We used *C. gibelio* as the reference genome and removed all repeats. Illuminua reads of two *C. auratus*, six *C. gibelio* and one *C. carpio* (Supplementary Table 16 and 17) were mapped to the *C. gibelio* genome using BWA (Version 0.7.12-r1039)[6]. Using the genome-wide mean reads depth (*m*) as a criterion, sites with reads depths between 0.5*m* and 2*m* were retained for subsequent analysis. Then, *C. gibelio*-specific mapped non-coding regions with uniform coverage of reads and lengths greater than 100 bp were defined as new non-coding elements. We investigated the functions of the closest genes within 10 Kb of both sides of new non-coding elements by GO enrichment analysis and found that many of these genes were associated with meiosis.

Supplementary Note 6 | Loss of heterozygosity analysis

For loss-of-heterozygosity (LOH) analysis, one female individual of G_4 generation of clone F[18] was selected to construct a *C. gibelio* clonal line by reproducing successive four generations via gynogenesis. We sequenced 11 individuals (~ 48× depth for each sample) from the offspring of the gynogenetic line and called SNPs of each individual as the method in "Resequencing-based ploidy analysis". To minimize both false-negative and false-positive calls, we used the following criteria to process SNPs and identify LOH sites in the SNP set identified in the 11 offsprings from the gynogenetic pedigree: (1) Filter the non-triploid chromosomes (Chr1B, 6A and 22A of *Cg*-F1 in Supplementary Fig. 13). (2) Trimorphic SNP sites that have three different bases account for only 0.26% (34,916) of the total SNP sites, and the rest are dimorphic, of which the depth of one type of base is usually twice of the other type

(referred to as the minor allele), indicating most SNPs were singletons (minor allele) in the first parental mother of the gynogenetic pedigree. Therefore, we used these dimorphic SNPs for following analyses. (3) SNPs with a minimum average of 20× coverage and a maximum coverage of 80× were maintained. (4) Sites directly adjacent to small insertion-deletion mutations were filtered to avoid false-positive inferences created by misalignment. (5) For each SNP site of one individual, the coverage depth of minor allele ≥ 5× was considered as heterozygous site, and ≤ 1× was considered as homozygous site. (6) If SNPs in any individual with a coverage depth of minor allele > 1× and < 5× were considered as ambiguous sites and filtered from the SNP set. (7) LOH sites were only called when they were heterozygous in some individuals but became unambiguously homozygous in one or more individual(s). Finally, 64,246 LOH sites were obtained from a total of 9,780,732 SNP sites. To verify these LOH sites, we used Sanger sequencing to examine 101 randomly selected LOH sites. PCR primers were designed using Primer 5 based on *C. gibelio* reference genome sequence, capturing approximately 300 bp flanking the LOH locus on both sides. Every amplified fragment was cloned, and then 30 clones were picked to sequence for determining the genotype of the SNP site. 97 LOH sites were verified.

Unlike in diploids where LOH (homozygous) SNP sites are continuous, every LOH block may contain both LOH SNP sites and still heterozygous SNP sites (referred to as non-LOH) in triploids (Extended Data Fig. 4a). Given the discontinuity of LOH sites, we next restricted our search to contiguous tracts of LOH sites, where the length of the tract was 100 kb. We considered the first LOH site found on a tract to

be part of a possible LOH region and iteratively extended the region if a next LOH site was found within 100 kb to the previous LOH site. The tract length of each LOH region was calculated from the interval midpoint between the first LOH site and upstream non-LOH SNP site to interval midpoint between the last LOH site and downstream non-LOH SNP site. Furthermore, we filtered the LOH regions with only one LOH site presenting in a single individual.

After identifying LOH sites and regions, we then moved to filter deletion regions. (1) We plotted the distribution of the average normalized read depth for SNP sites in each LOH region, and it showed two peaks around 49× (triploid) and 31× (diploid) (Extended Data Fig. 4b). (2) We plotted the distribution for the average frequency of minor allele in each LOH region, and the results also showed two peaks around 0.33× (triploid) and 0.42× (diploid) (Extended Data Fig. 4c). (3) LOH regions with an average read depth >40× and average frequency of minor allele <0.37 at the same time were considered as gene conversion regions (triploid) (p-value < 0.05 in one or more lines, binomial test). Eventually, we obtained the candidate gene conversion regions that contained 61,014 LOH sites (95.0% of total LOH sites) in the 11 individuals of the gynogenetic pedigree.

Finally, we analyzed the identified gene conversion regions based on the unique SNP-converted pattern in triploids. As shown in Extended Data Fig. 4a, after a gene deletion, there could be two SNP sites: 1/3 of SNP sites show LOH and the rest remain heterozygous with similar read depths for each allele (like in diploids); however, after a conversion, there could be three types of SNP sites: homozygous

converted sites which result in LOH in this region, heterozygous converted sites where the donor allele is minor allele before conversion, and converted sites that look unchanged where the donor allele has same base as the recipient allele before conversion but heterozygous with the minor allele, and their ratios should show a pattern of 1/3:1/3:1/3 if a conversion region is long enough. Accordingly, we calculated the ratios of the three types of SNP sites in the candidate gene conversion regions; exactly, each type approximately occupied 1/3 of total SNP sites in a conversion region (Extended Data Fig. 4d). Moreover, we phased the blocks by comparing homologous SNP sites between individuals that did or did not experience gene conversion, where SNP genotyping was determined by the read coverages of its two base statuses (Supplementary Fig. 18). Since gene conversion is a unidirectional DNA modification from one haplotype to another, the donor and recipient alleles at each SNP site can be inferred respectively in the gynogenetic pedigree, and thereby the three haplotypes will be phased. As expected, the phasing blocks (Fig. 5f and Extended Data Fig. 5) present a well-defined SNP pattern for gene conversion in triploid (Extended Data Fig. 4a). Therefore, these data indicated that the identified gene conversion regions are basically reliable.

The rate of LOH (per locus per generation) was calculated following the method in Omilian et al.[19] using the equation $\lambda = h / (L \times i \times T)$, where h is the number of observed LOH sites, L is the number of lines, i is the number of total considered informative sites, and T is the number of generations for lines. The rates of LOH, GC and GD were calculated respectively.

Supplementary Note 7 | Assembly of male-specific supernumerary sequences

In order to obtain male-specific supernumerary sequences, we sequenced a male individual *C. gibelio* (F strain) using Illumina sequencing technology. A total of 333 Gb reads were mapped to the reference genome of *C. gibelio* with a mapping rate of 99.23%. The unmapped reads were then used to assemble possible male-specific regions using platanus v1.2.4. Finally, we obtained 33 Kb sequences, with a N50 of 16.6 Kb. Only one gene (*tufm*) was found in the assembled sequence. Unfortunately, we noticed that this gene is highly similar to the copy in *Streptococcus*, indicating this is most likely from the contamination during sampling and sequencing process.

References

1 Ranallo-Benavidez, T. R., Jaron, K. S. & Schatz, M. C. GenomeScope 2.0 and Smudgeplot for reference-free profiling of polyploid genomes. *Nat. Commun.* **11**, 1432 (2020).

2 Kokot, M., Długosz, M. & Deorowicz, S. KMC 3: counting and manipulating k-mer statistics. *Bioinformatics* **33**, 2759-2761 (2017).

3 Marçais, G. & Kingsford, C. A fast, lock-free approach for efficient parallel counting of occurrences of k-mers. *Bioinformatics* **27**, 764-770 (2011).

4 Liu, B. *et al.* Estimation of genomic characteristics by analyzing k-mer frequency in de novo genome projects. *arXiv: Genomics* (2013).

5 Luo, R. *et al.* SOAPdenovo2: an empirically improved memory-efficient short-read de novo assembler. *GigaScience* **1**, 18 (2012).

6 Li, H. Aligning sequence reads, clone sequences and assembly contigs with BWA-MEM. *arXiv* (2013).

7 Li, H. *et al.* The sequence alignment/map format and SAMtools. *Bioinformatics* **25**, 2078-2079 (2009).

8 Quinlan, A. R. & Hall, I. M. BEDTools: a flexible suite of utilities for comparing genomic features. *Bioinformatics* **26**, 841-842 (2010).

9 Simao, F. A., Waterhouse, R. M., Ioannidis, P., Kriventseva, E. V. & Zdobnov, E. M. BUSCO: assessing genome assembly and annotation completeness with single-copy orthologs.

Bioinformatics **31**, 3210-3212 (2015).

10 Chen, D. *et al.* The evolutionary origin and domestication history of goldfish (*Carassius auratus*). *Proc. Natl. Acad. Sci. U. S. A.* **117**, 29775-29785 (2020).

11 Wen, M. *et al.* Sex chromosome and sex locus characterization in goldfish, *Carassius auratus* (Linnaeus, 1758). *BMC Genomics* **21**, 552 (2020).

12 Yang, Z. H. PAML 4: Phylogenetic analysis by maximum likelihood. *Mol. Biol. Evol.* **24**, 1586-1591 (2007).

13 Kim, D., Landmead, B. & Salzberg, S. L. HISAT: a fast spliced aligner with low memory requirements. *Nat. Methods* **12**, 357-360 (2015).

14 Pertea, M. *et al.* StringTie enables improved reconstruction of a transcriptome from RNA-seq reads. *Nat. Biotechnol.* **33**, 290-295 (2015).

15 Wang, K. *et al.* Morphology and genome of a snailfish from the Mariana Trench provide insights into deep-sea adaptation. *Nat. Ecol. Evol.* **3**, 823-833 (2019).

16 Frampton, M. & Houlston, R. Generation of artificial FASTQ files to evaluate the performance of next-generation sequencing pipelines. *PLoS ONE* **7**, 0049110 (2012).

17 Su, T. T. & Stumpff, J. Promiscuity rules? The dispensability of cyclin E and Cdk2. *Sci. STKE* **2004**, pe11 (2004).

18 Chen, F. *et al.* Stable genome incorporation of sperm-derived DNA fragments in gynogenetic clone of gibel carp. *Mar. Biotechnol.* **22**, 54-66 (2020).

19 Omilian, A. R., Cristescu, M. E., Dudycha, J. L. & Lynch, M. Ameiotic recombination in asexual lineages of *Daphnia*. *Proc. Natl. Acad. Sci. U. S. A.* **103**, 18638-18643 (2006).

Additional Supplementary Figures

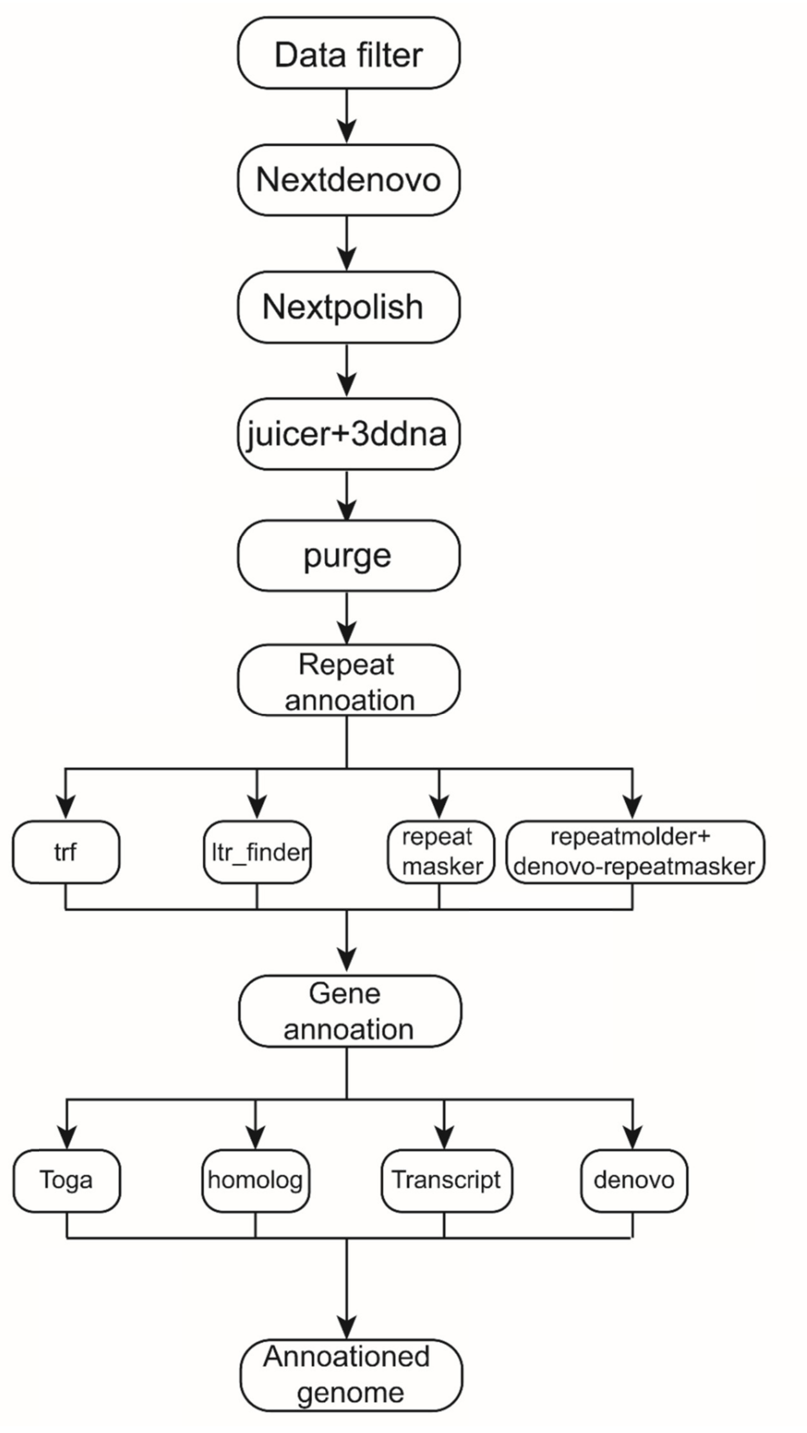

Supplementary Figure 1 | Assembly strategy of genomes of *C. auratus* and *C. gibelio*.

Please refer the method section for detail introduction.

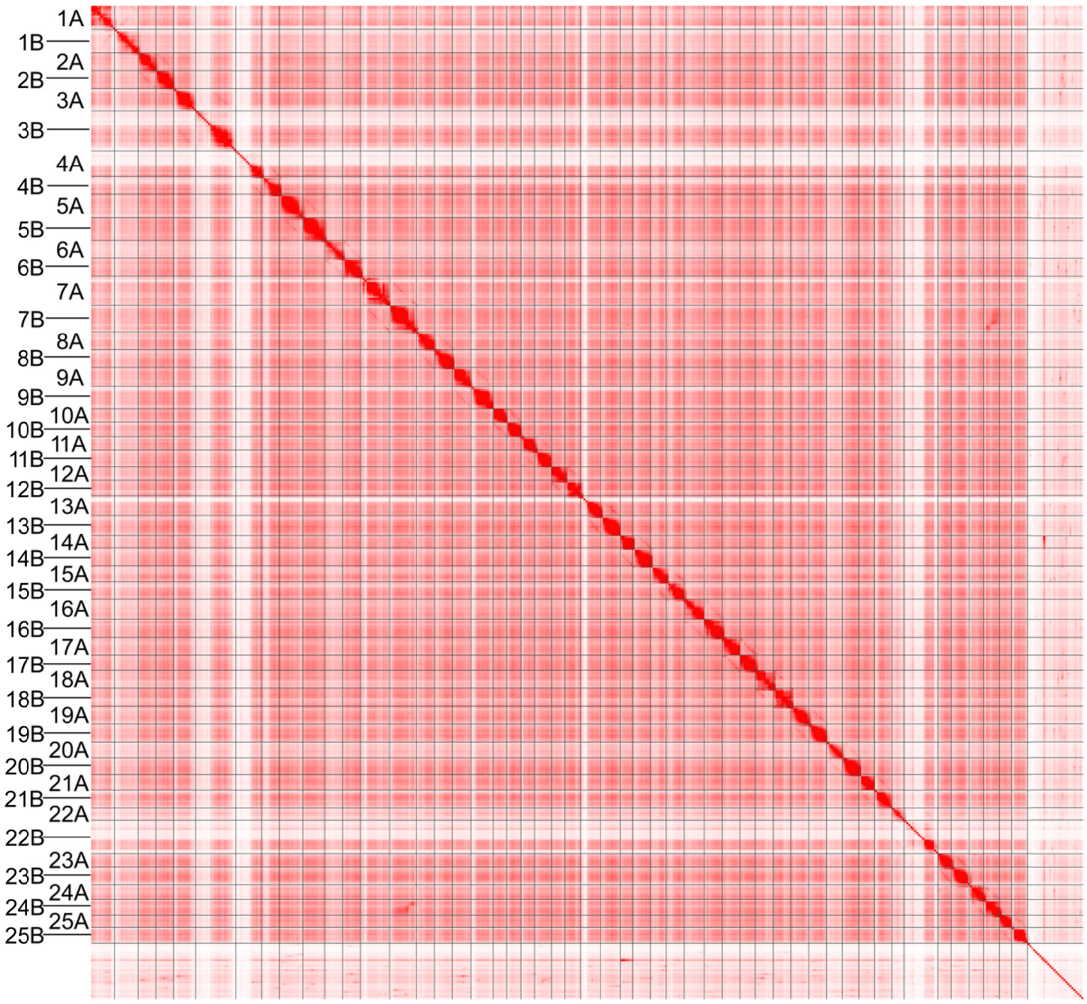

Supplementary Figure 2 | The interaction Hi-C data map of *C. gibelio*.

A deeper color (red) represents a stronger level of interaction. Two points can be drawn from this figure, 1) the chromosome boundaries can be clearly delineated, and 2) the interactions between subgenome A and B are weak, indicating that they have been diverged for a considerable period of time.

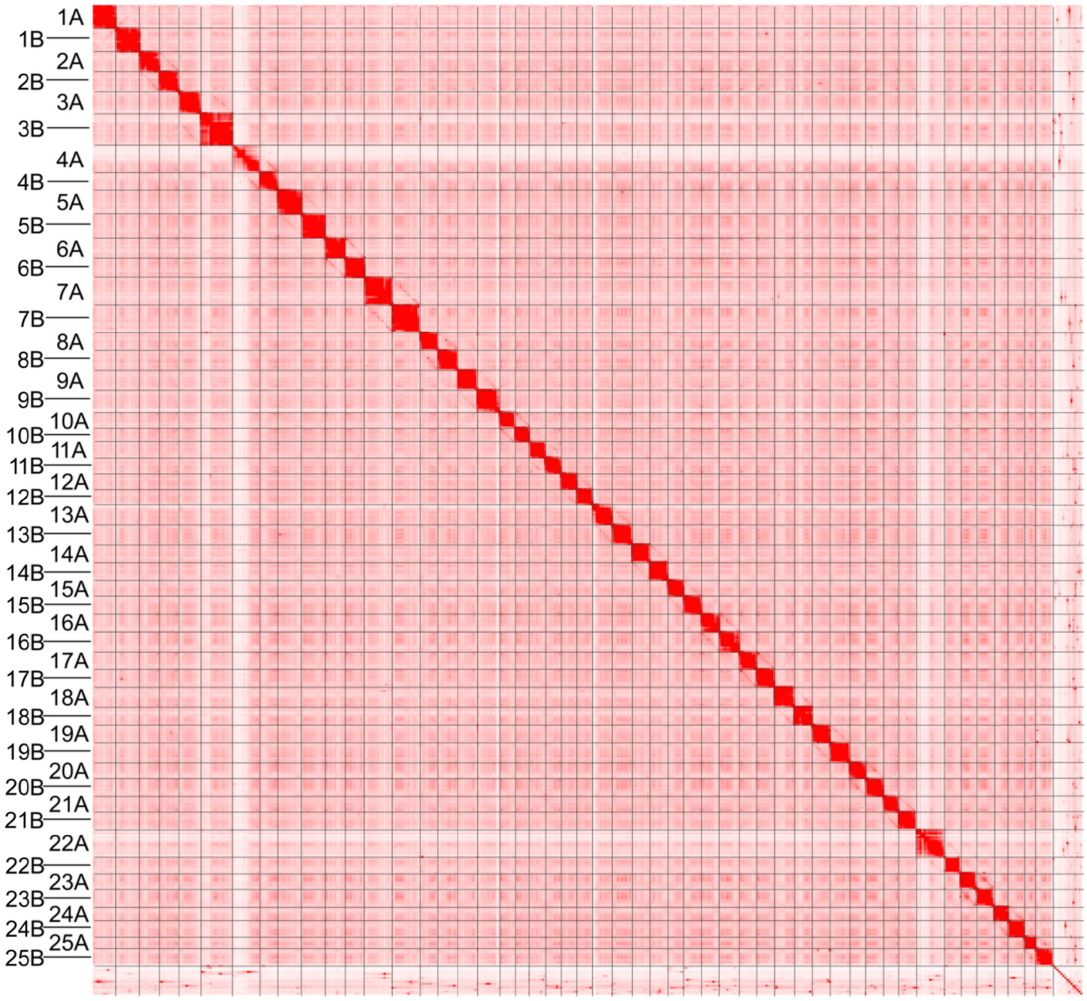

Supplementary Figure 3 | The interaction Hi-C data map of *C. auratus*.

A deeper color (red) represents a stronger level of interaction. Two points can be drawn from this figure, 1) the chromosome boundaries can be clearly delineated, and 2) the interactions between subgenome A and B are weak, indicating that they have been diverged for a considerable period of time.

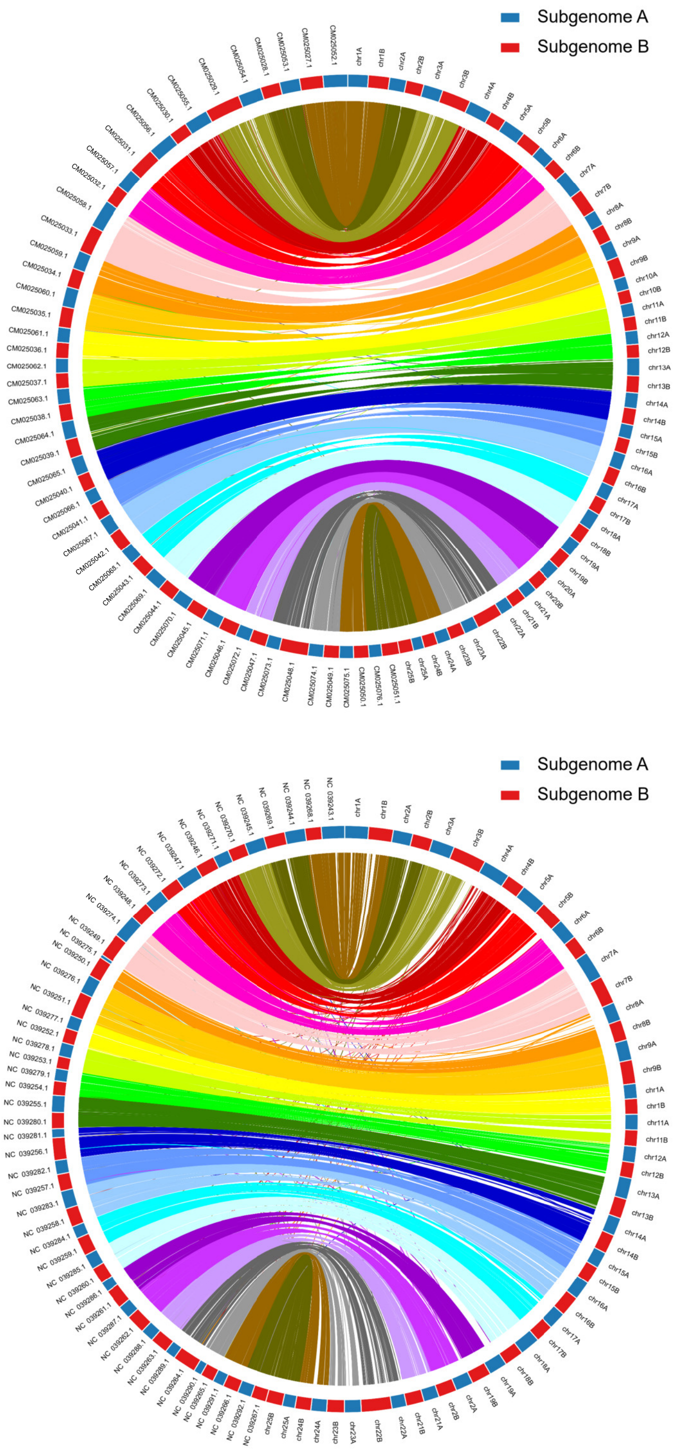

Supplementary Figure 4 | Syntenies between genomes of *C. auratus* of this study (right, chr prefix) and previous versions (left, above GCA_014332655.1 from Chen et al. 2020 and below GCF_003368295.1 from Chen et al. 2019).

This result indicates that the delineation of subgenome A and B in this study is fully consistent with the previous versions of the genome assemblies of *C. auratus*.

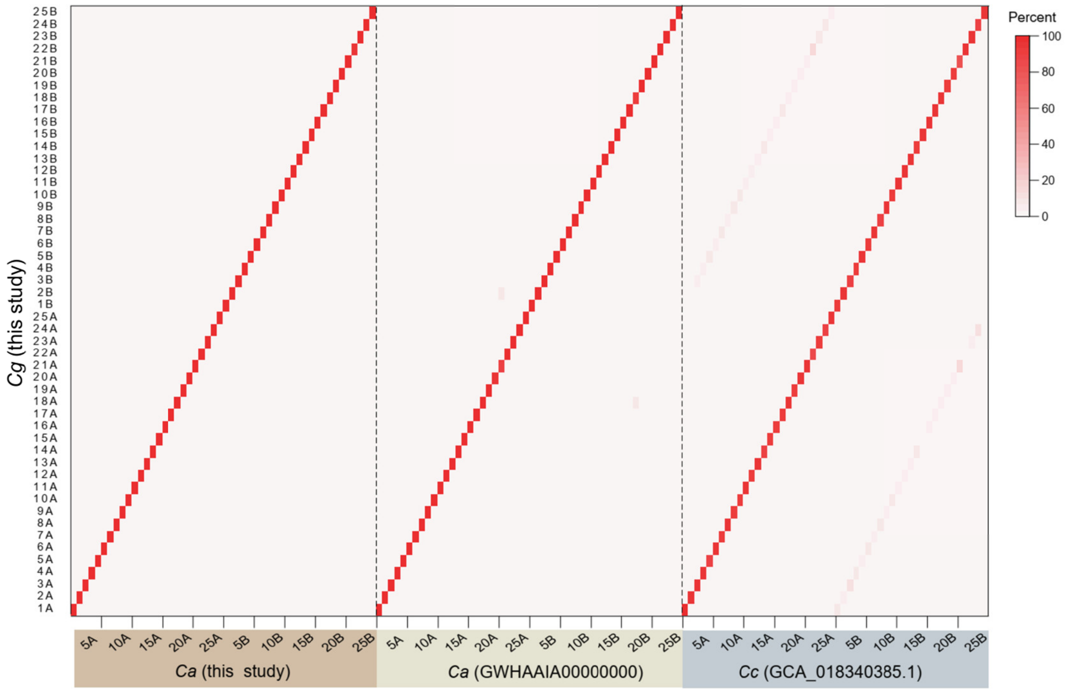

Supplementary Figure 5 | Percentage of reciprocal best hit orthologs for each pair of chromosomes of *C. gibelio* to *C. auratus* (this study), goldfish (GWHAAIA00000000 from Luo et al. 2020), and common carp (GCA_018340385.1 from Li et al. 2021).

Red to white indicate high to low similarity values, respectively.

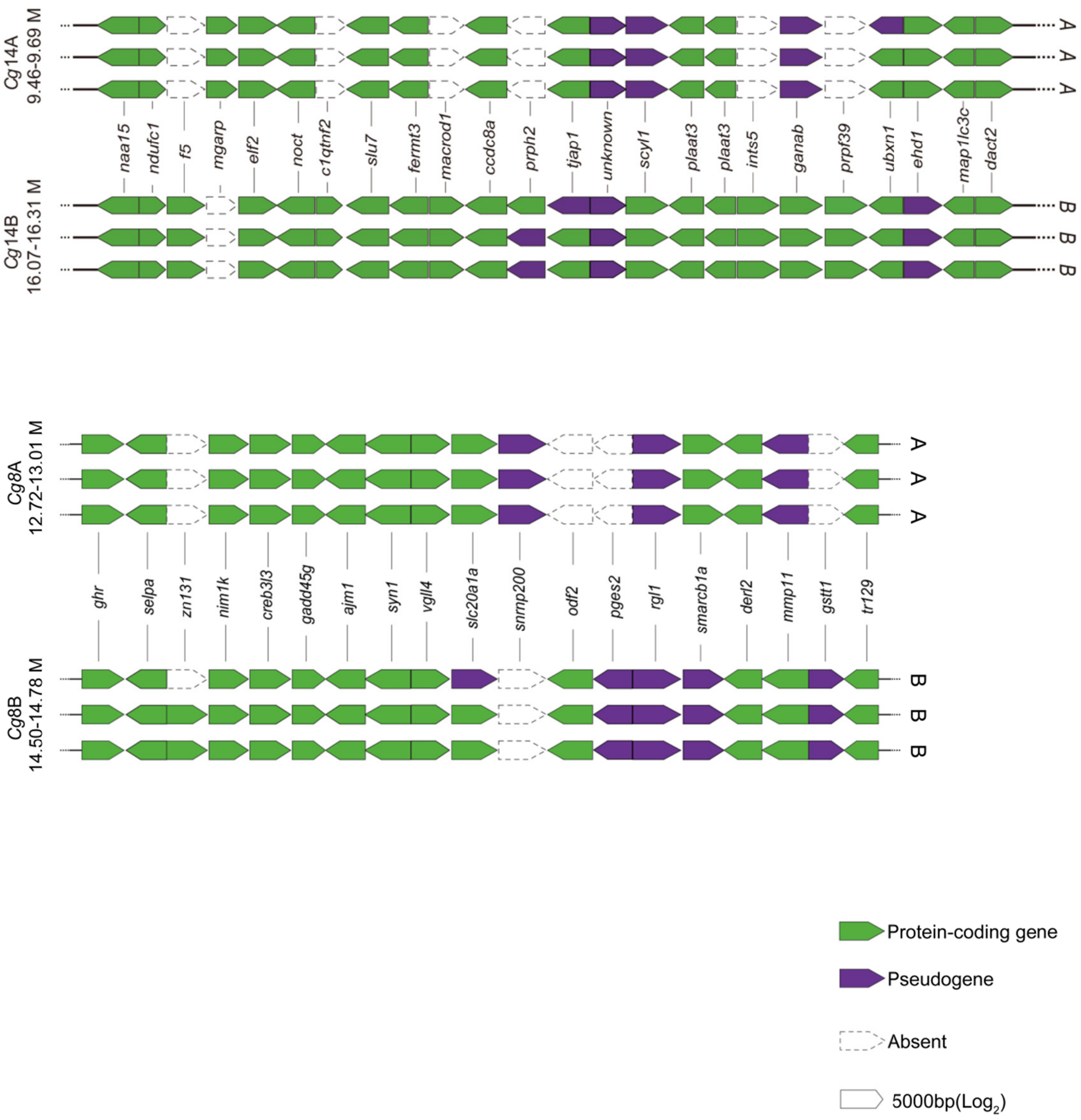

Supplementary Figure 6 | Homeologous genes and homologous alleles within two examples of BAC phasing blocks.

This result demonstrates that there are three haplotypes for *C. gibelio*, and there is also genetic diversity among the three haplotypes.

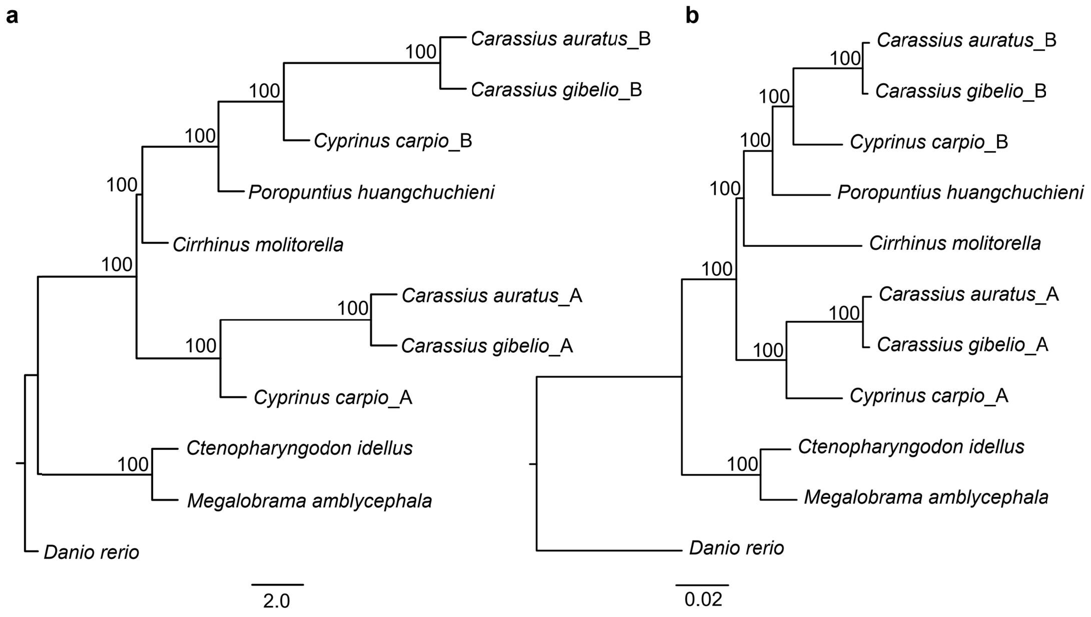

Supplementary Figure 7 | Phylogenetic relationships of *C. auratus* and *C. gibelio* subgenomes based on ASTRAL (a) and IQ-TREE (b).

The two phylogenetic trees have a same topology and subgenome B are clustered with P. *huangchuchieni* and *C. molitorella*.

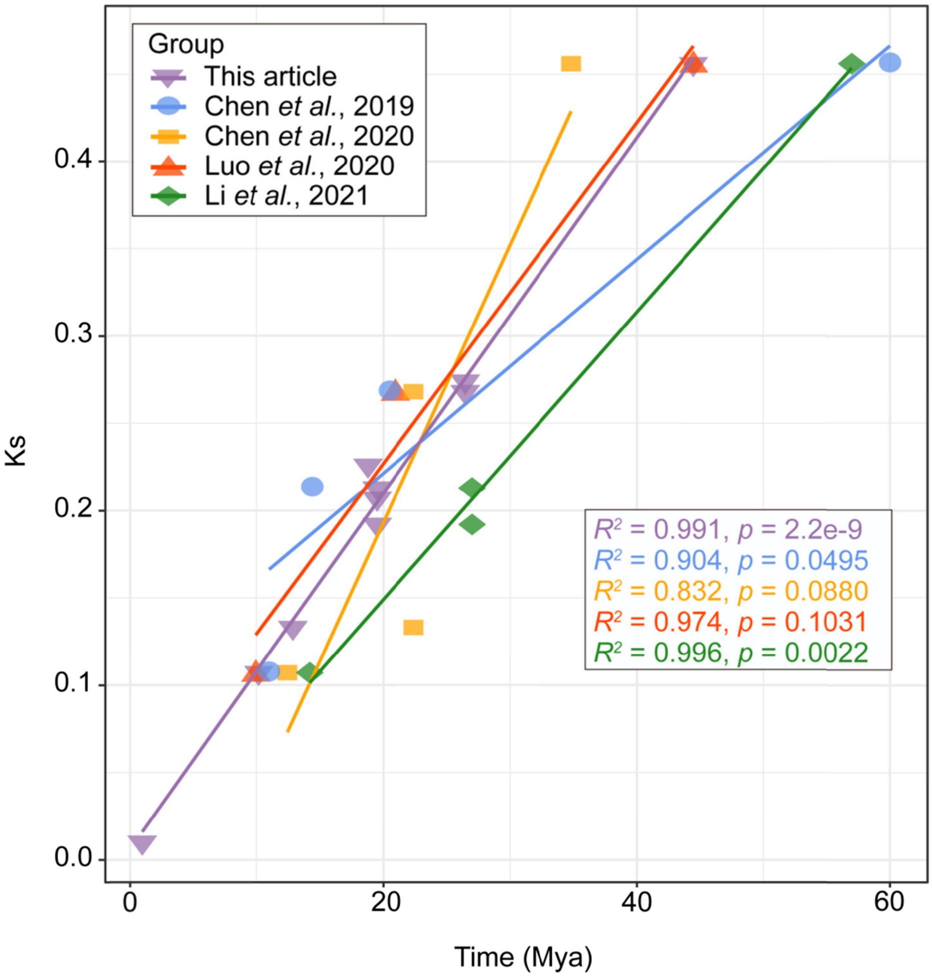

Supplementary Figure 8 | Correlation between divergence time and *Ks* from different studies.

Each point in the figure represents a differentiated node, where the horizontal coordinate refers to the estimated divergence time in each study (distinguished by color) and the vertical coordinate refers to the *Ks* value between two branches of this node. When points from different studies had the same *Ks* value, we put them in the same differentiated node. We applied Pearson's correlation coefficient analysis to calculate the R^2 value and *p*-value for divergence times (from different studies) and *Ks* distribution.

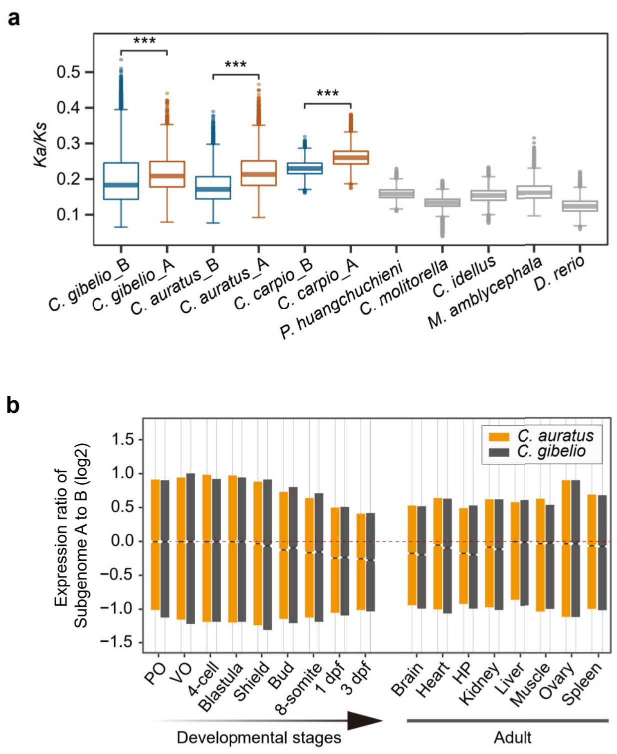

Supplementary Figure 9 | Dominance of subgenome B.

a, Different degrees of selective relaxation of subgenome A and B exhibited by *Ka*/*Ks* ratios using orthologous genes. To assess the level of *Ka/Ks* in each species, we performed 10,000 random samples. For each sampling, 150 genes were randomly selected from all orthologous genes for concatenation, and their *Ka/Ks* as a whole were calculated in each species. The difference in *Ka/Ks* between species was calculated using paired students' t-tests (two-sided without multiple adjustment). The *p*-values are: *C. gibelio*_A-*C. gibelio*_B: 0, *C. auratus*_A-*C. auratus*_B: 0, *C. carpio*_A-*C. carpio*_B: 0. A lower *Ka*/*Ks* indicates a higher level of purifying selection in subgenome B, which suggests that subgenome B is more likely to be the dominant genome. The line in the middle of each boxplot represents the median of the dataset; the upper and lower edges of boxplot indicate the third quartile and the first quartile, respectively; and the line extending from the edge is 1.5 times the interquartile range. Small dots indicate outliers. "***" indicates a *p*-value < 0.001. A lower *Ka/Ks* indicates a higher level of purifying selection in subgenome B, which suggests that subgenome B is more likely to be the dominant genome. **b,** Biased gene expression in the development stages and adult tissues of *Carassius*. The biased expression of B genes gradually increases in later stages of embryonic development, indicating that the subgenome B should play a more important functional role than subgenome A; thus, the B genes may have higher expression levels. HP, hypothalamus and pituitary; PO, pre-vitellogenic oocytes; VO, vitellogenic oocytes; dpf, day after fertilization.

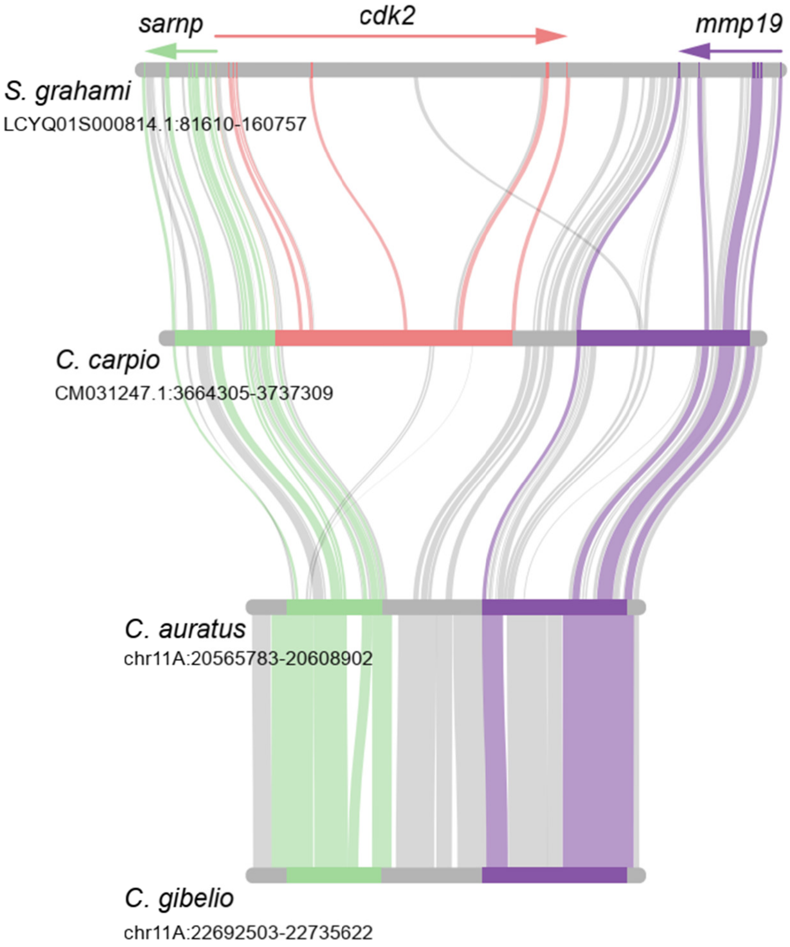

Supplementary Figure 10 | Gene loss showed by synteny chart.

The gene *cdk2* can be found in *S. grahami* and *C. carpio*, but lost in *C. auratus* and *C. gibelio*.

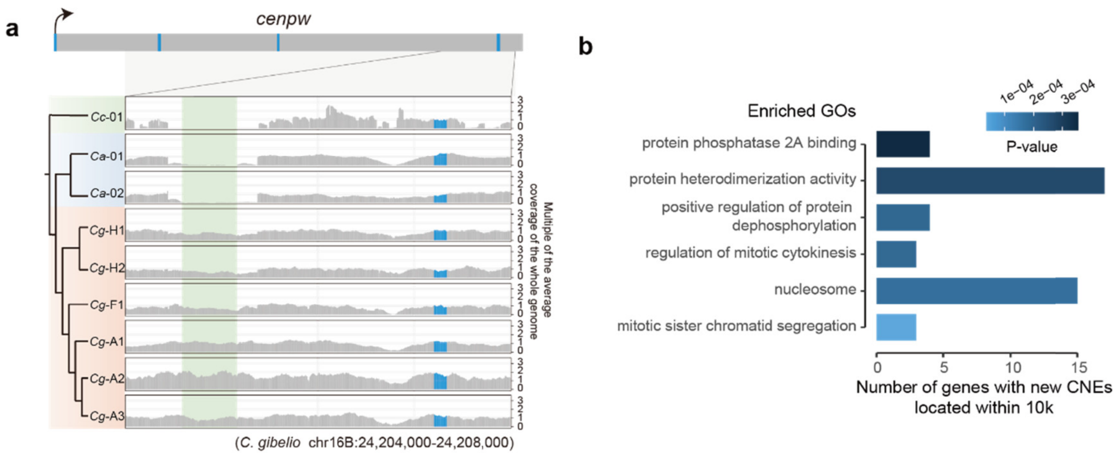

Supplementary Figure 11 | Newly originated elements in *C. gibelio*.

a, Example of newly originated elements in *C. gibelio*. The upper panel shows the gene structure of *cenpw*, and the bar chart below represents the relative read depth for each individual mapped to the *C. gibelio* genome. Blue and green indicate coding and *C. gibelio*-specific element regions, respectively. The arrow indicates transcription direction. **b**, Enriched gene ontologies (GOs) of the genes with new elements located within 10 kb.

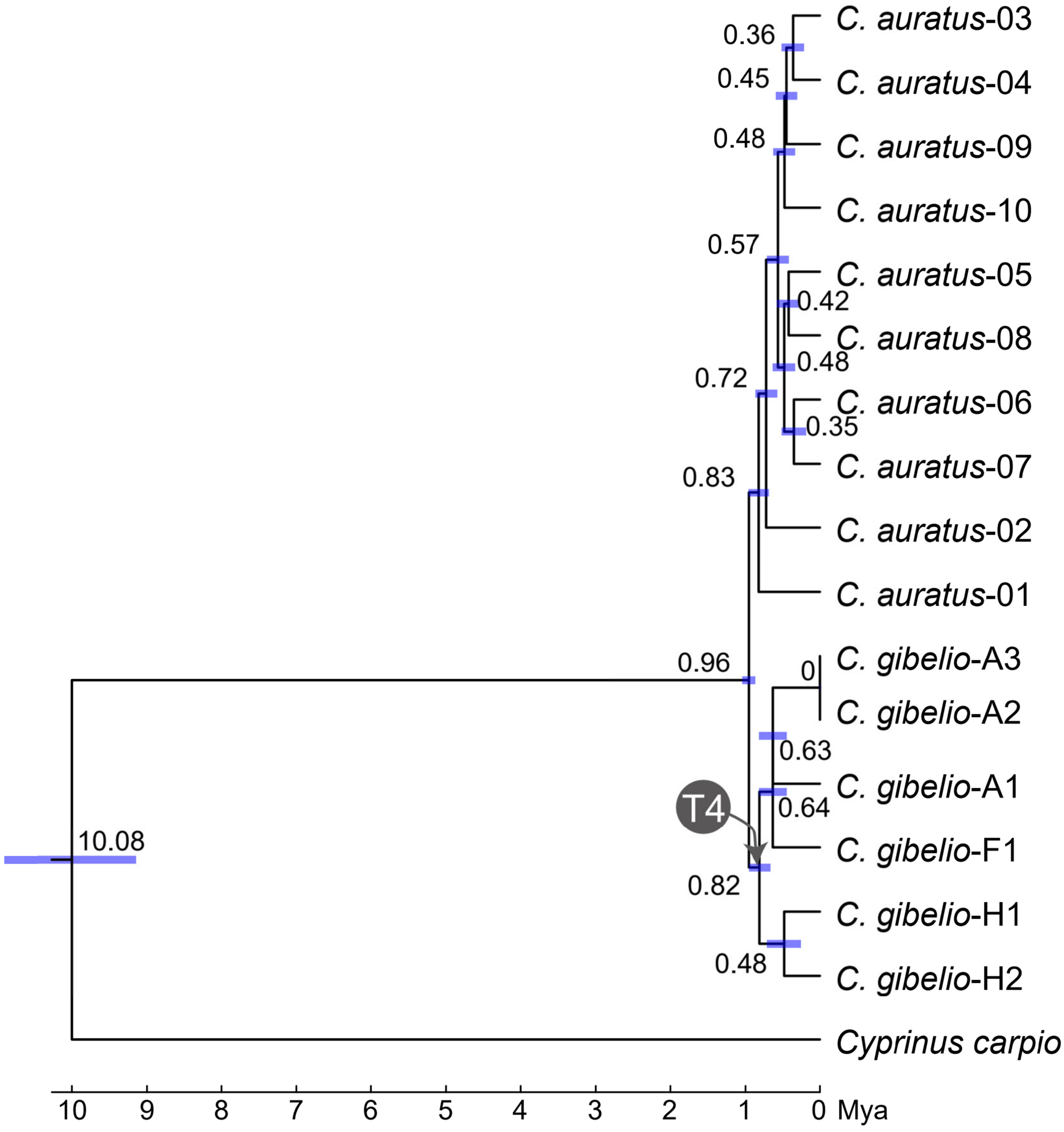

Supplementary Figure 12 | Divergence time of three *C. gibelio* strains based on SNPs from resequencing data.

The divergence time of three *C. gibelio* strains (T4) is indicated by the large grey circle. The blue rectangles represent the 95% confidence intervals.

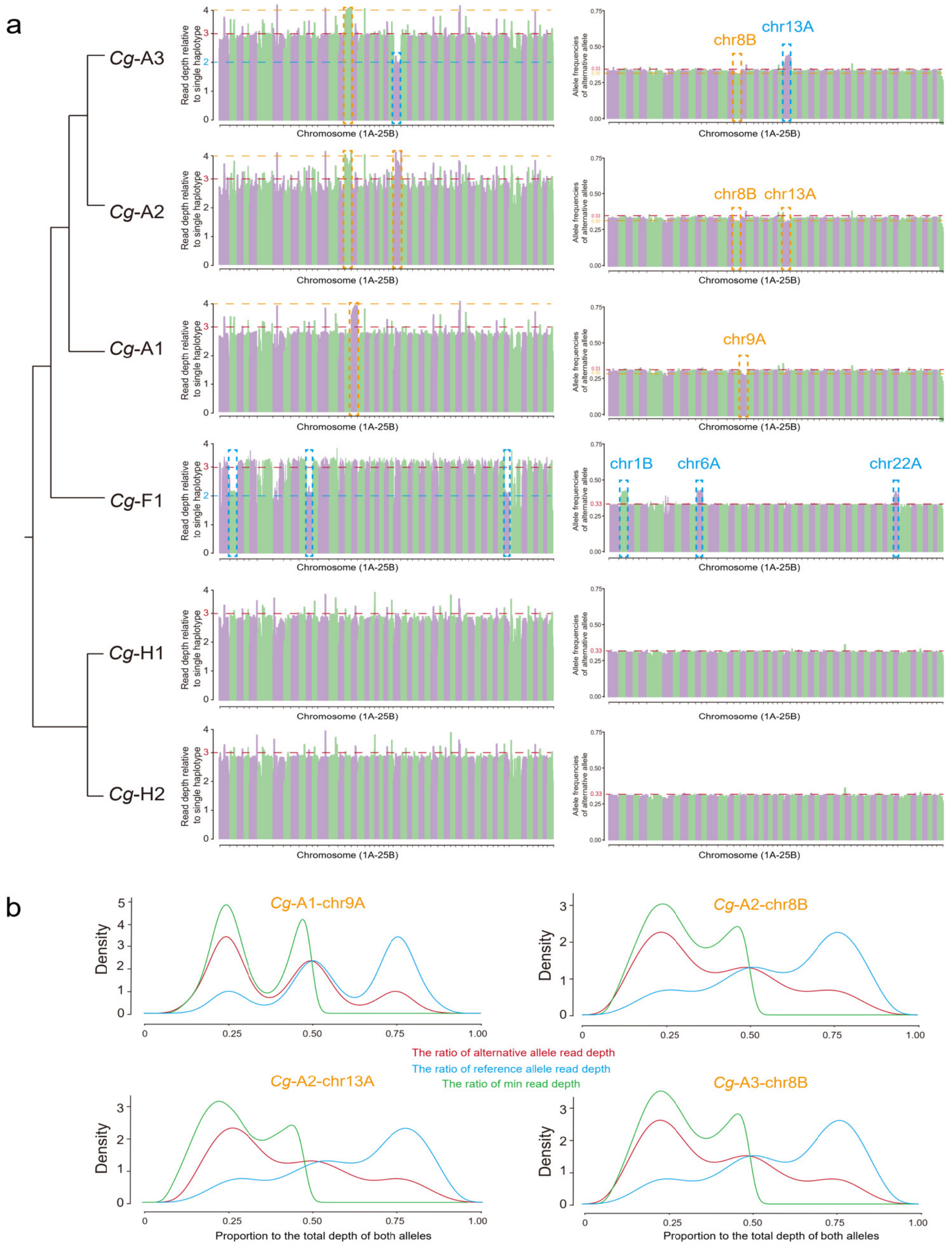

Supplementary Figure 13 | The ploidy differences of some chromosomes in different individuals of *C. gibelio*.

a, Left panel is the read depth relative to the single haplotype in each chromosome, and right panel is the allele frequencies of alternative alleles in each chromosome. The blue and orange boxes with dash line are the cases of chromosome loss and gain respectively. Each color block represents a chromosome. **b,** The distribution density of the read depth of each SNP site in the duplicated chromosome (orange) in panel A. The x-axis represents the ratio of the read depth of a single SNP site divided by the sum of the read depths of all loci.

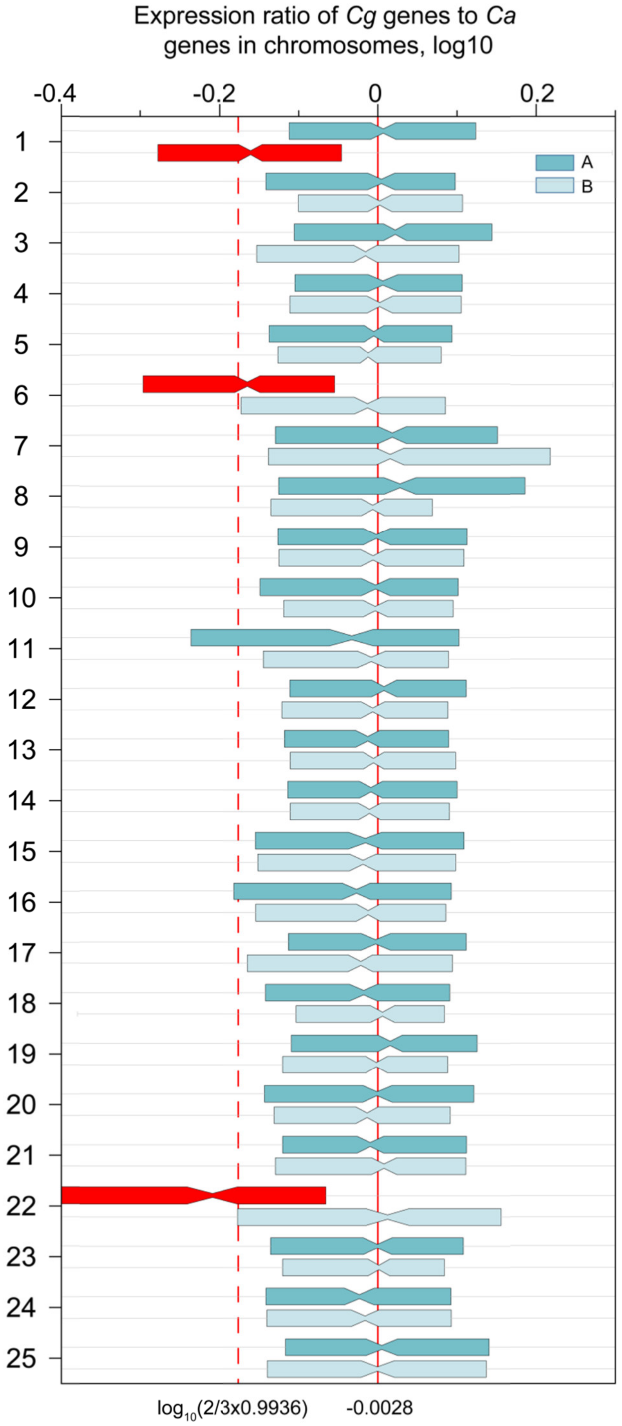

Supplementary Figure 14 | Expression ratio of homologous genes of *C. gibelio*/*C. auratus* along each chromosome, zoomed in to show medians.

Disomic chromosomes are marked with red. The median of the expression ratio of all genes between *C. gibelio* and *C. auratus* is 0.9936 ($\log_{10}0.9936 = -0.0028$).

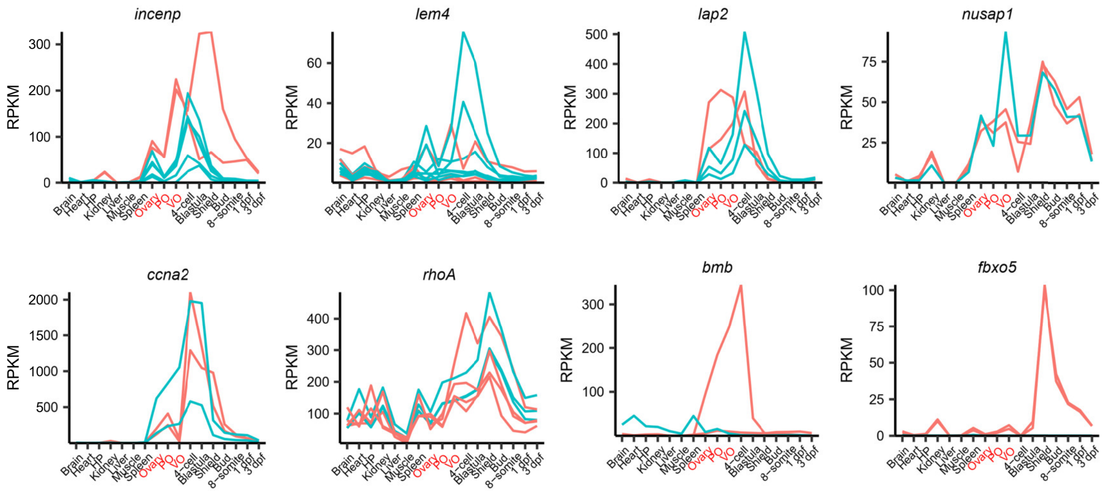

Supplementary Figure 15 | Expression pattern of the eight expanded gene families related to oocyte formation in *C. gibelio*.

The red lines represent the old genes of the two *Carassius* species. The green lines represent the newly evolved copies in *C. gibelio*. HP, hypothalamus and pituitary; PO, pre-vitellogenic oocytes; VO, vitellogenic oocytes; dpf, days post-fertilization.

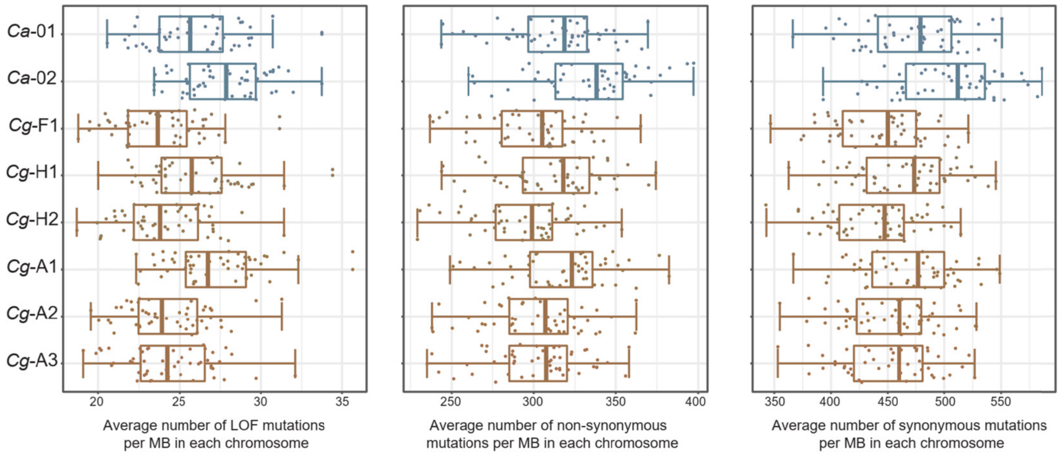

Supplementary Figure 16 | Average number of different types of mutations in different individuals.

Left, the ID of each individual. The horizontal coordinate refers to the average number of mutations (fixed in each individual) per individual within each megabase (MB). For each individual there are 50 points, where each point represents the average of the number of mutations per 1MB on one chromosome. The line in the middle of each boxplot represents the median of the dataset; the upper and lower edges of boxplot indicate the third quartile and first quartile, respectively; and the line extending from the edge is 1.5 times the interquartile range. Small dots indicate outliers.

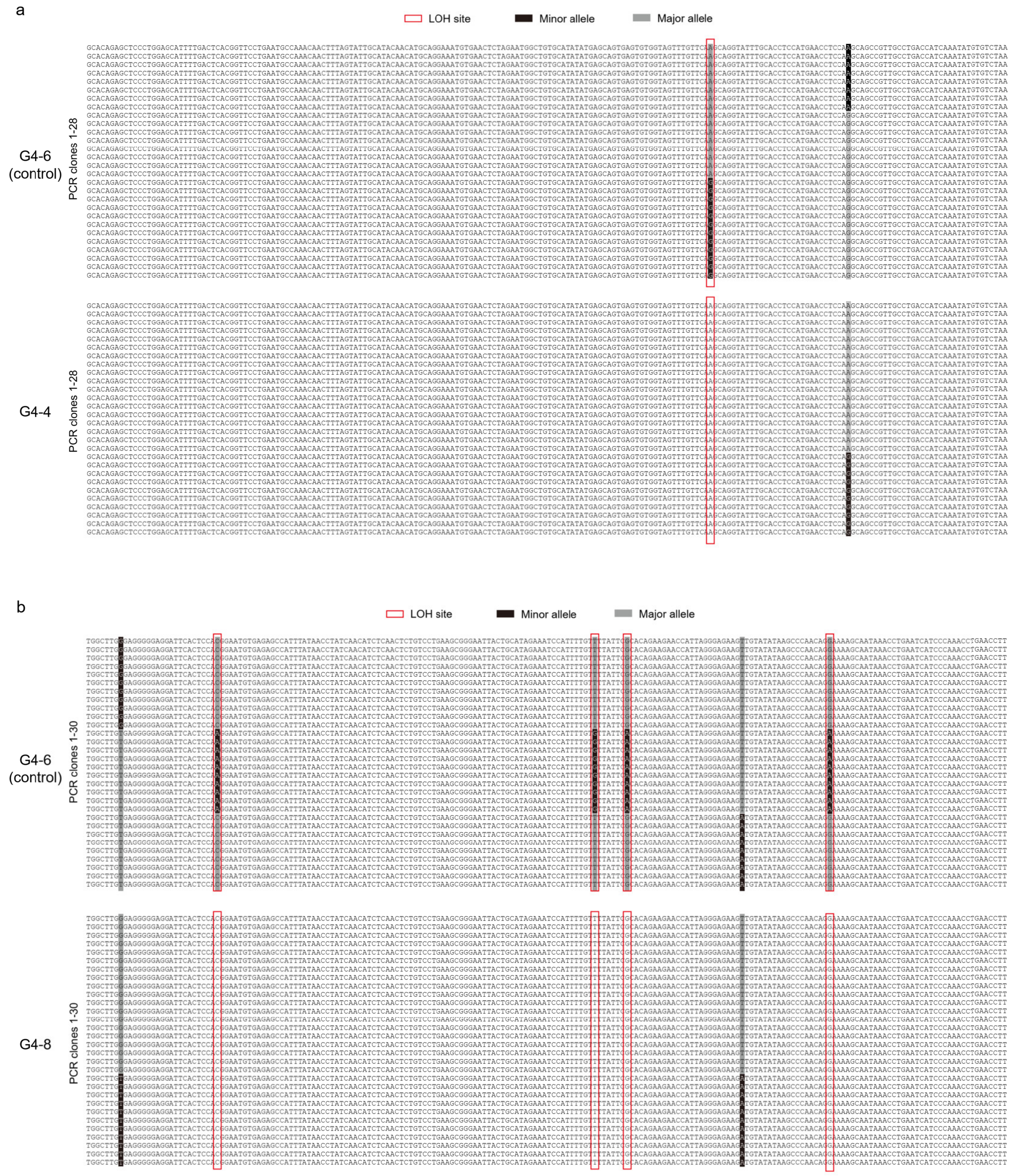

Supplementary Figure 17 | Verification for LOH sites at chromosome A2 of G4-4 (a) and A12 of G4-8 (b).

The sequences of PCR clones for each sample are aligned. G4-6 is used as a control without gene conversion. Loss of heterozygosity (LOH) site is indicated with red frame. Major and minor alleles of SNP are shadowed by grey and black, respectively.

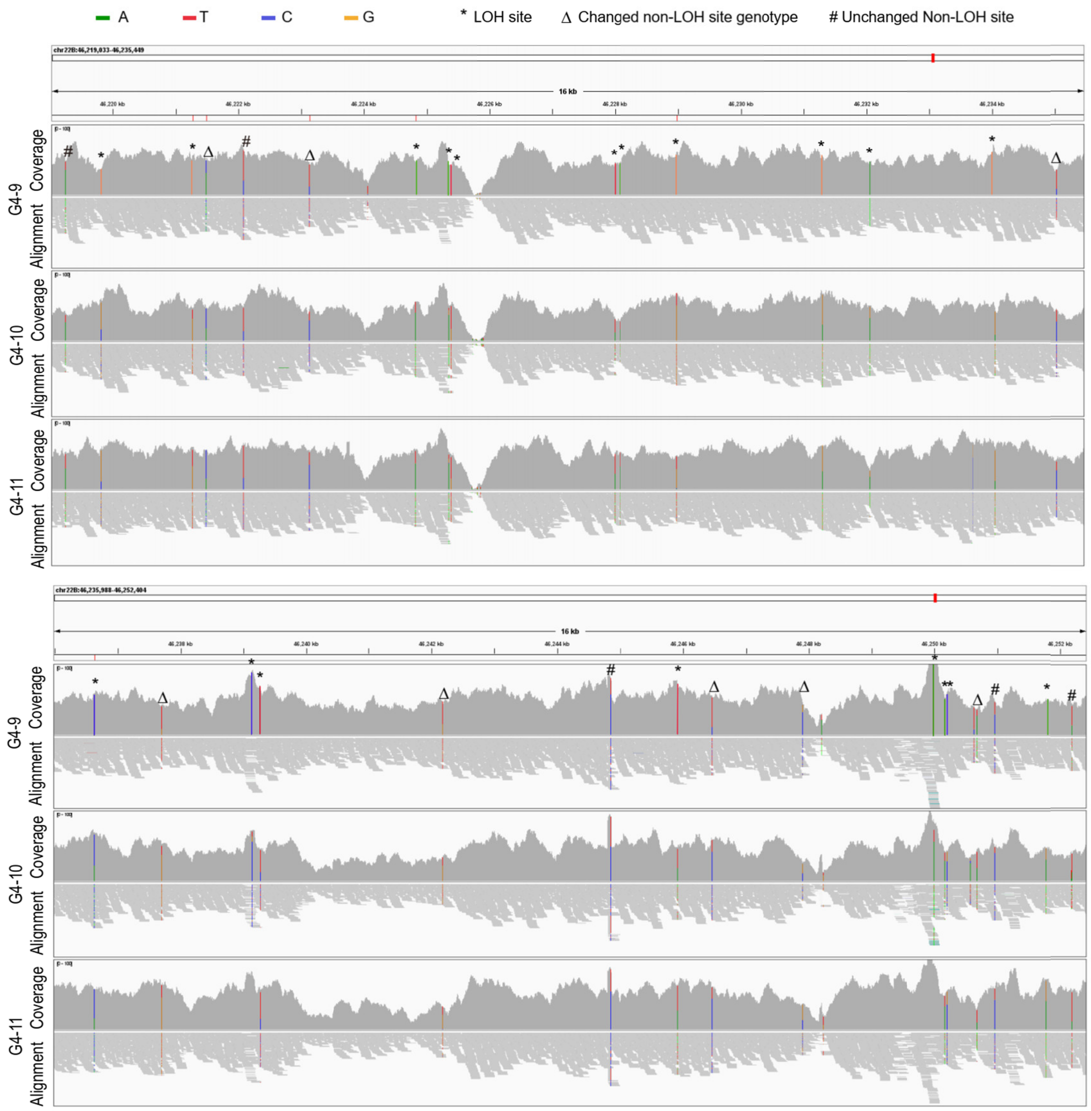

Supplementary Figure 18 | Read coverage for a block in the gene conversion region shown in Fig. 5e.

The same positions of G4-9, G4-10, and G4-11 individuals are visualized using IGV software. In each sample, the top panel shows the read coverage based on the aligned reads presented in the bottom panel. Four bases (ATCG) at SNP sites are presented with different colors. *, LOH site. Δ, Non-LOH site with changed genotype after conversion. #, Non-LOH site with unchanged genotype after conversion.

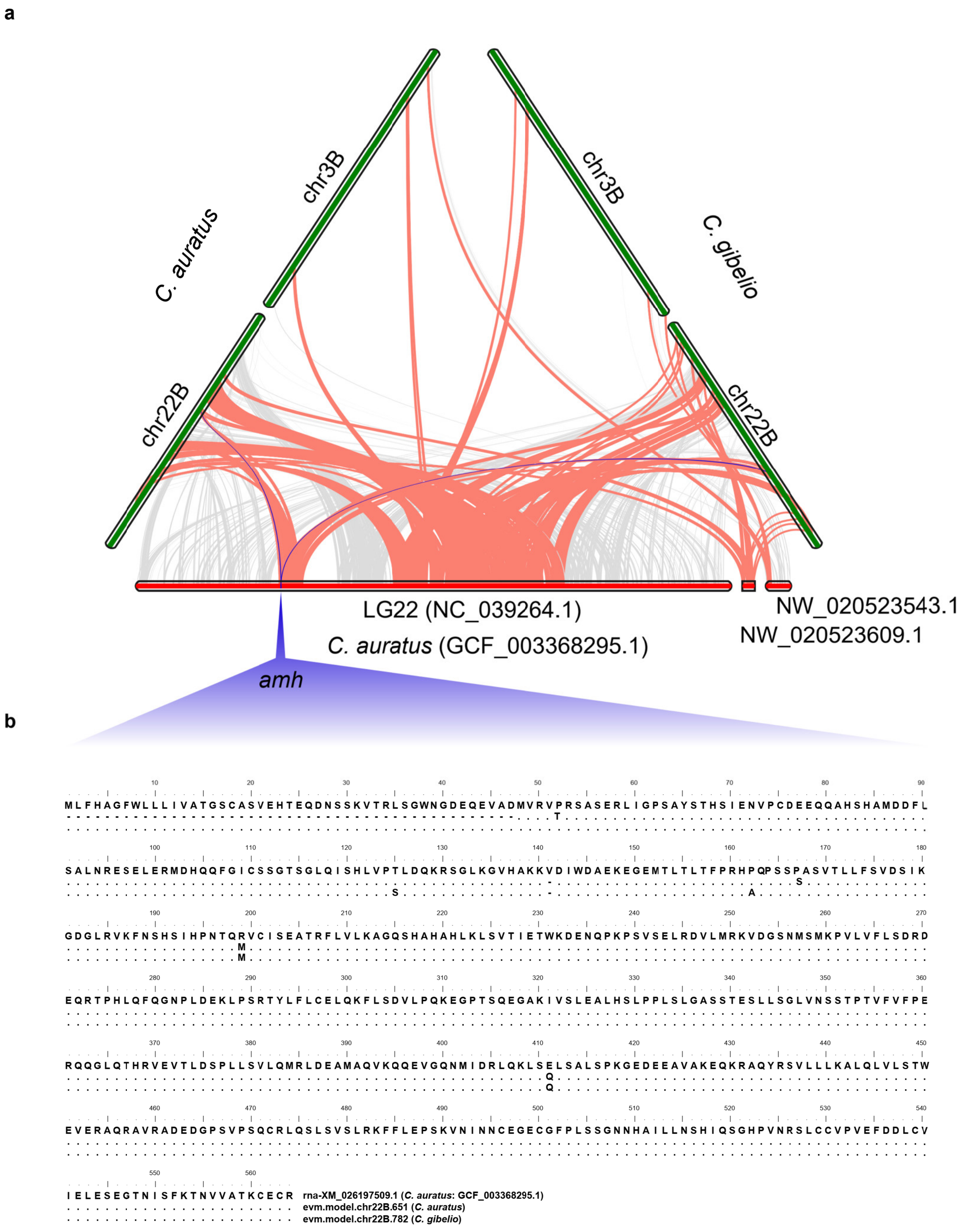

Supplementary Figure 19 | Alignments of the sex determination region and potential master sex gene *amh* of *C. auratus*.

a, Synteny alignment of the sex chromosomes between different versions of *C. auratus* genomes and the *C. gibelio* genome. The red bars indicate the sex chromosome regions. **b,** Protein sequence alignment of Amh. The results indicate that it is largely conserved between *C. auratus* and *C. gibelio*.

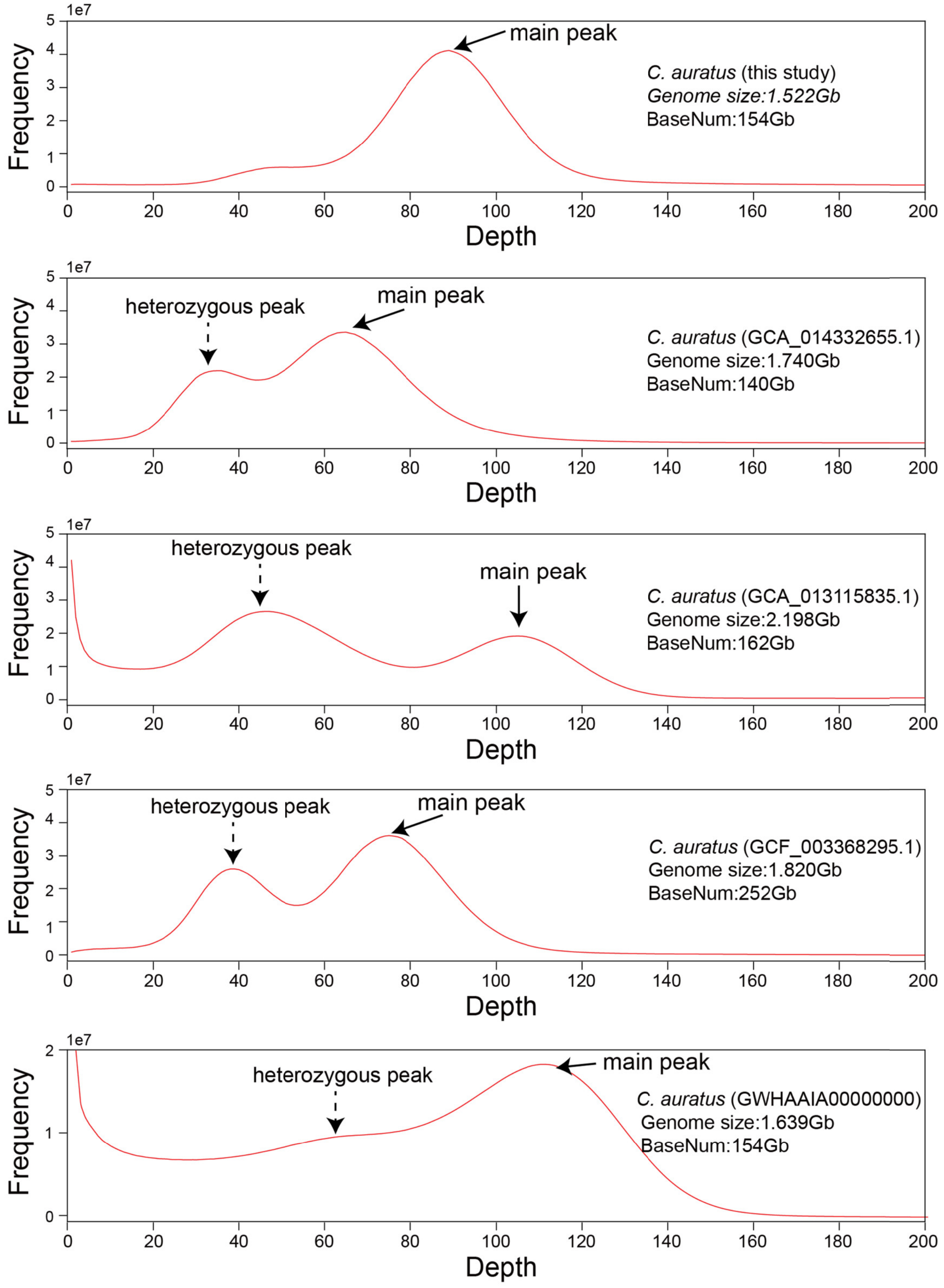

Supplementary Figure 20 | Assessment of heterozygous sequences in different versions of *C. auratus* genome using purge_dups.

These sequences were largely resulted from assembly redundancy. The horizontal coordinate refers to the read depth, and the vertical coordinate refers to the read occurrence frequency.

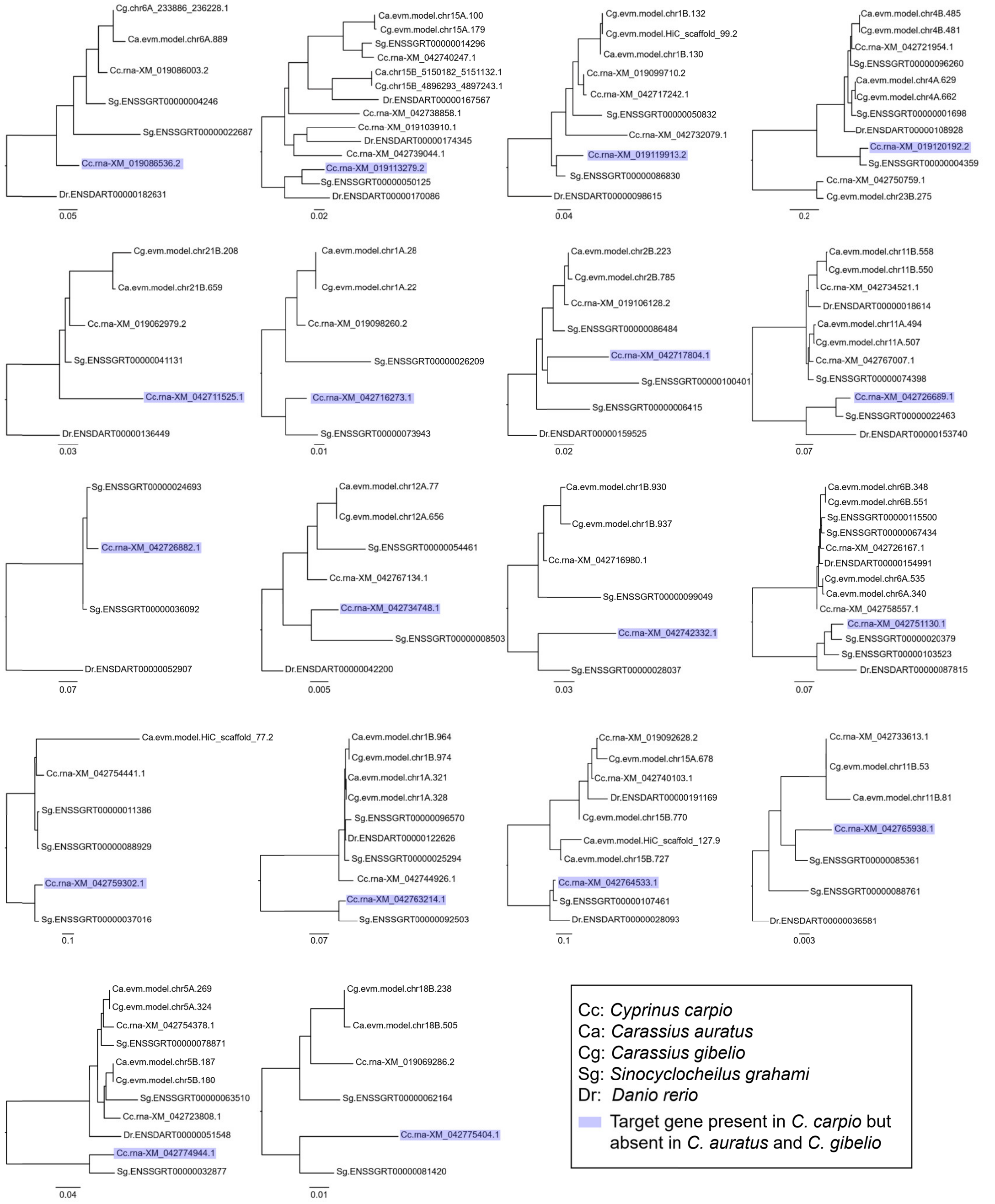

Supplementary Figure 21 | The gene tree for identify lineage specific gene loss in *Carassius*.

The gene with blue shadow indicate the target gene present in *C. carpio* but are absent in *C. auratus* and *C. gibelio*. It can be observed that the target gene has orthologues in *S. graham* but no orthologous in both *Carassius* species.

Additional Supplementary Tables

Supplementary Table 1 | PacBio sequencing data for *C. auratus* and *C. gibelio*

Species	Technical platform	Data size (Gb)	N50 (bp)
C. auratus	Pacbio	155	15,284
C. gibelio	Pacbio	313	14,286

Supplementary Table 2 | Hi-C sequencing data for *C. auratus* and *C. gibelio*

Species	Library	Read Num	Read length	Data Size (bp)
C. auratus	M1	1,543,033,248	150	231,454,987,200
Total		1,543,033,248	150	231,454,987,200
C. gibelio	CL100025299_L01	420,301,178	50	21,015,058,900
	CL100025299_L02	360,399,650	50	18,019,982,500
	CL100025878_L01	376,831,278	50	18,841,563,900
	CL100025878_L02	374,100,028	50	18,705,001,400
	CL100033765_L01	416,175,436	50	20,808,771,800
	CL100033765_L02	418,462,186	50	20,923,109,300
Total		2,366,269,756	50	118,313,487,800

Supplementary Table 3 | Illumina sequencing data for *C. auratus* and *C. gibelio*

Species	Read length (bp)	Illumina insert size (bp)	Raw data		Clean data	
			Data (Mb)	Sequence depth (X)	Data (Mb)	Sequence depth (X)
C. auratus	270_270	150	142,278	94.85	126,390	84.26
	270_270	150	141,356	94.24	125,595	83.73
	Total	----	283,634	189.09	251,985	167.99
C. gibelio	100_100	170	44,154	29.44	41,663	27.78
	100_100	500	32,943	21.96	30,312	20.21
	100_100	800	33,260	22.17	29,632	19.75
	49_49	2000	50,787	33.86	34,903	23.27
	49_49	5000	32,896	21.93	22,696	15.13
	49_49	10000	26,648	17.77	21,969	14.65
	49_49	20000	19,767	13.18	14,317	9.54
	49_49	40000	38,059	25.37	11,281	7.52
	Total	----	278,515	185.68	206,773	137.85

Supplementary Table 4 | Illumina transcriptome sequencing data statistics

Species	Sample	Tissues	Total reads	Total map	Total map rate	Uniq. map	Uniq. map rate
C. auratus	Adult tissues	heart	98,061,212	66,739,849	68.10%	60,838,298	62.00%
		liver	94,502,224	58,288,768	61.70%	50,650,524	53.60%
		kidney	99,494,294	73,345,791	73.70%	66,165,937	66.50%
		muscle	87,056,771	56,984,973	65.50%	49,310,428	56.60%
		ovary	104,986,909	71,141,621	67.80%	58,283,238	55.50%
		hypothalamus and pituitary	95,462,969	69,681,096	73.00%	64,301,701	67.40%
		other brains	107,500,027	77,856,154	72.40%	72,269,199	67.20%
	Embryos	4-cell	92,761,155	68,331,398	73.70%	59,596,348	64.20%
		blastula	81,925,327	59,818,433	73.00%	52,102,580	63.60%
		gastrula	75,167,463	53,471,367	71.10%	44,316,860	59.00%
		bud	107,120,885	79,045,340	73.80%	66,911,808	62.50%
		8-somite	94,281,258	68,631,398	72.80%	58,312,069	61.80%
		1 dpf (day after fertilization)	86,756,997	63,968,107	73.70%	53,410,456	61.60%
		3 dpf	83,575,502	59,761,784	71.50%	50,946,971	61.00%
	Oocytes	pre-vitellogenic oocytes	44,728,504	30,450,890	68.10%	25,399,242	56.80%
		vitellogenic oocytes	44,726,122	32,085,853	71.70%	28,387,424	63.50%
C. gibelio	Adult tissues	heart	95,549,447	69,120,566	72.30%	61,130,158	64.00%
		liver	108,894,253	71,074,927	65.30%	58,846,601	54.00%
		kidney	95,354,377	73,144,201	76.70%	61,450,940	64.40%
		muscle	92,678,783	58,116,723	62.70%	45,577,669	49.20%
		ovary	92,173,778	65,175,416	70.70%	38,946,017	42.30%
		hypothalamus and pituitary	103,754,354	76,503,105	73.70%	68,283,553	65.80%

	other brains	92,732,324	69,450,537	74.90%	61,892,802	66.70%
Embryos	4-cell	88,416,034	66,014,840	74.70%	55,957,759	63.30%
	blastula	101,061,195	76,180,684	75.40%	65,160,783	64.50%
	gastrula	92,139,303	67,491,505	73.20%	55,086,326	59.80%
	bud	85,704,498	63,341,989	73.90%	52,999,082	61.80%
	8-somite	85,087,623	62,948,145	74.00%	53,040,855	62.30%
	1 dpf	82,541,256	60,028,677	72.70%	49,832,620	60.40%
	3 dpf	87,302,785	64,121,235	73.40%	54,280,928	62.20%
Oocytes	pre-vitellogenic oocytes	44,768,571	31,965,644	71.40%	24,589,691	54.90%
	vitellogenic oocytes	44,723,737	35,579,843	79.60%	30,181,437	67.50%

Supplementary Table 5 | Genome size estimation using SOAPec v2.01.

Species	Parameter	Estimated genome size (Gb)
C. auratus	-k 17	1.44
C. auratus	-k 19	1.48
C. auratus	-k 21	1.51
C. auratus	-k 23	1.54
C. auratus	-k 25	1.53
C. gibelio	-k 17	1.49
C. gibelio	-k 19	1.54
C. gibelio	-k 21	1.50
C. gibelio	-k 23	1.56
C. gibelio	-k 25	1.52

Supplementary Table 6 | Genome assemblies of *C. auratus* and *C. gibelio*

Species	Contig N50 (bp)	Scaffold N50 (bp)	Assembly size (bp)	Busco
C. auratus	3,890,216	29,195,226	1,522,436,151	98.20%
C. gibelio	1,709,526	28,762,065	1,589,834,947	98.10%

Supplementary Table 7 | Genome assemblies of each chromosome in *C. auratus* and *C. gibelio*

Chromosome lengths of *C. auratus*			Chromosome lengths of *C. gibelio*		
**Chromosome ID**	**GC content**	**Length (bp)**	**Chromosome ID**	**GC content**	**Length (bp)**
chr1A	0.37	35,386,190	chr1A	0.37	40,897,126
chr1B	0.37	36,562,877	chr1B	0.37	36,702,745
chr2A	0.38	29,847,804	chr2A	0.38	28,137,009
chr2B	0.37	30,271,480	chr2B	0.37	28,700,003
chr3A	0.38	33,475,191	chr3A	0.38	34,360,084
chr3B	0.38	50,118,171	chr3B	0.38	64,159,174
chr4A	0.38	40,215,424	chr4A	0.38	42,573,681
chr4B	0.38	28,104,874	chr4B	0.38	29,259,740
chr5A	0.38	38,253,659	chr5A	0.38	36,903,395
chr5B	0.37	36,161,242	chr5B	0.37	35,066,000
chr6A	0.37	30,939,686	chr6A	0.37	29,499,805
chr6B	0.37	29,573,676	chr6B	0.37	28,218,437
chr7A	0.38	41,370,417	chr7A	0.38	45,653,107
chr7B	0.38	42,068,215	chr7B	0.38	43,450,849
chr8A	0.38	27,700,533	chr8A	0.38	27,457,764
chr8B	0.37	30,422,264	chr8B	0.37	30,398,283
chr9A	0.38	30,268,488	chr9A	0.38	29,602,934
chr9B	0.37	35,413,027	chr9B	0.37	34,409,320
chr10A	0.37	22,862,535	chr10A	0.38	22,316,396
chr10B	0.37	23,375,766	chr10B	0.37	23,430,943
chr11A	0.38	23,395,214	chr11A	0.38	23,560,082

chr11B	0.37	24,479,626	chr11B	0.37	23,153,646
chr12A	0.37	24,042,631	chr12A	0.37	24,812,728
chr12B	0.37	23,631,608	chr12B	0.37	24,235,296
chr13A	0.38	31,450,662	chr13A	0.38	32,508,620
chr13B	0.37	29,417,698	chr13B	0.37	29,838,850
chr14A	0.38	26,914,525	chr14A	0.37	21,547,070
chr14B	0.38	28,775,669	chr14B	0.38	28,762,065
chr15A	0.38	24,531,501	chr15A	0.38	24,602,895
chr15B	0.38	26,664,337	chr15B	0.38	27,590,285
chr16A	0.38	28,399,711	chr16A	0.38	30,712,257
chr16B	0.37	30,504,434	chr16B	0.38	30,610,988
chr17A	0.38	26,344,876	chr17A	0.38	26,464,210
chr17B	0.37	27,353,385	chr17B	0.37	27,036,235
chr18A	0.38	30,088,908	chr18A	0.38	29,498,950
chr18B	0.38	28,619,747	chr18B	0.38	28,119,819
chr19A	0.38	27,243,412	chr19A	0.38	28,337,080
chr19B	0.38	29,195,226	chr19B	0.38	28,154,658
chr20A	0.37	26,118,538	chr20A	0.37	25,488,214
chr20B	0.37	27,371,858	chr20B	0.37	27,122,856
chr21A	0.38	22,963,375	chr21A	0.37	22,480,024
chr21B	0.37	26,431,221	chr21B	0.38	28,952,007
chr22B	0.38	45,457,829	chr22A	0.38	19,964,608
chr22A	0.38	22,536,000	chr22B	0.37	54,135,076
chr23A	0.38	24,885,537	chr23A	0.38	22,944,178
chr23B	0.38	26,272,494	chr23B	0.38	28,493,337
chr24A	0.38	23,430,752	chr24A	0.37	23,250,111

chr24B	0.37	23,634,917	chr24B	0.37	23,800,854
chr25A	0.38	18,940,634	chr25A	0.38	19,684,772
chr25B	0.37	24,967,067	chr25B	0.37	25,117,204
Total	0.38	1,476,454,911	Total	0.38	1,502,175,770

Supplementary Table 8 | Summary of annotation in *C. auratus* and *C. gibelio*

Species	Gene number	Average gene length (bp)	Average CDS length (bp)	Average exons per gene	Average exon length (bp)	Repeat of genome
C. auratus	44,283	18,542.22	1,609.75	9.44	170.61	44.61%
C. gibelio	45,249	20,169.19	1,639.32	9.59	170.90	45.85%

Supplementary Table 9 | Gene annotation of *C. auratus* and *C. gibelio*

Species	Type		Gene number	Gene length	Cds length
C. auratus		*Danio rerio*	41,428	13,038.65	170.52
	homolog	*Oryzias latipes*	36,875	12,521.51	170.22
		Gasterosteus aculeatus	37,142	12,413.94	169.59
	De novo	augustus	37,504	24,951.71	175.69
	RNA-seq		33,886	19,137.49	163.25
	evm		44,283	18,542.22	170.61
C. gibelio		*Danio rerio*	43,402	13,000.94	170.75
	homolog	*Oryzias latipes*	38,317	12,263.57	170.19
		Gasterosteus aculeatus	38,642	12,185.45	169.57
	De novo	augustus	39,744	25,123.79	175.50
	RNA-seq		34,334	18,997.60	163.61
	evm		45,249	20,169.19	170.90

Supplementary Table 10 | Repeat annotation of *C. auratus* and *C. gibelio*

Species	Type	Repeat Size (bp)	% of genome
C. auratus	Trf	68,238,418	4.48
	Repeatmasker	251,068,053	16.49
	Proteinmask	142,280,950	9.35
	De novo	570,918,989	37.50
	Total	679,224,193	44.61
C. gibelio	Trf	75,367,997	4.74
	Repeatmasker	265,620,065	16.71
	Proteinmask	150,423,288	9.46
	De novo	617,677,558	38.85
	Total	728,981,203	45.85

Supplementary Table 11 | Summary of TE contents in *C. auratus* and *C. gibelio*

Species	Type	Length (bp)	% in genome
C. auratus	DNA	238,234,847	15.65
	LINE	66,418,806	4.36
	SINE	11,992,130	0.79
	LTR	70,986,972	4.66
	Other	60,914,239	4.00
	Satellite	55,243,274	3.63
	Simple repeat	5,670,965	0.37
	Unknown	139,545,370	9.17
	Total	570,918,989	37.50
C. gibelio	DNA	244,125,246	15.36
	LINE	66,471,816	4.18
	SINE	8,852,687	0.56
	LTR	75,755,529	4.76
	Other	69,296,540	4.36
	Satellite	64,523,985	4.06
	Simple repeat	4,772,555	0.30
	Unknown	171,673,553	10.80
	Total	617,677,558	38.85

Supplementary Table 12 | Quality comparison among different versions of the genomes

Genome assembly version	Scaffolds number	Total length (bp)	Longest scaffold (bp)	N50 of scaffolds (bp)	N90 of scaffolds (bp)	Total contigs length (bp)	Longest contig (bp)	N50 of contigs (bp)	N90 of contigs (bp)	Length of 'N' (bp)	Percent of 'N'	Chromosomal assembled length (bp)	Chromosomal assembled portion (%)
C. auratus (this article)	231	1,522,436,151	50,118,171	29,195,226	23,375,766	1,522,212,175	21,324,548	3,890,216	584,006	223,976	0.0001	1,476,454,911	96.98%
C. gibelio (this article)	312	1,589,834,947	64,159,174	28,762,065	22,316,396	1,589,300,798	17,047,717	1,709,526	249,458	534,149	0.0003	1,502,175,770	94.49%
C. auratus (GCF_003368295.1)	6,216	1,820,635,050	37,185,075	22,763,433	86,816	1,820,410,350	9,630,712	821,153	73,670	224,700	0.0001	1,240,937,142	68.16%
C. auratus (GCA_014332655.1)	1,769	1,739,639,290	60,771,278	31,841,898	24,975,486	1,657,421,120	5,153,880	483,292	104,410	82,218,170	0.0473	1,654,030,761	95.08%
C. auratus (GWHAAIA00000000)	5,474	1,639,403,616	11,063,794	2,933,731	96,045	1,541,128,115	7,650,526	1,109,331	88,816	98,275,501	0.0599	NA	NA
C. auratus (GCA_013115835.1)	1,531,383	2,197,795,230	279,807	8,216	351	2,196,673,630	144,884	7,773	350	1,121,600	0.0005	NA	NA

Supplementary Table 13 | Comparison among different versions of the genomes using the results of BUSCO software (V5.2.2, actinopterygii_odb10 database, total 3,640 BUSCOs)

Genome assembly version	Complete	Complete and single	Complete and duplicate	Fragment	Missing
C. auratus (this article)	3,578 (98.30%)	1,634 (44.89%)	1,944 (53.41%)	21 (0.58%)	41 (1.13%)
C. gibelio (this article)	3,573 (98.16%)	1,634 (44.89%)	1,939 (53.27%)	27 (0.74%)	40 (1.10%)
C. auratus (GCF_003368295.1)	3,577 (98.27%)	1,357 (37.28%)	2,220 (60.99%)	26 (0.71%)	37 (1.02%)
C. auratus (GCA_014332655.1)	3,527 (96.90%)	1,607 (44.15%)	1,920 (52.75%)	37 (1.02%)	76 (2.09%)
C. auratus (GWHAAIA00000000)	3,542 (97.31%)	1,873 (51.46%)	1,669 (45.85%)	28 (0.77%)	70 (1.92%)
C. auratus (GCA_013115835.1)	2,959 (81.29%)	1,488 (40.88%)	1,471 (40.41%)	280 (7.69%)	401 (11.02%)

Supplementary Table 14 | Statistics on the number of different phylogenetic topology (top 10) of single-gene trees.

**Topology**	Total trees (3592)		High quality trees (698)	
	Count	Percent	Count	Percent
(((((((Carassius_auratus_B,Carassius_gibelio_B),Cyprinus_carpio_B),Poropuntius_huangchuchieni),Cirrhinus_molitorella),((Carassius_auratus_A,Carassius_gibelio_A),Cyprinus_carpio_A)),(Ctenopharyngodon_idellus,Megalobrama_amblycephala)),Danio_rerio)	888	0.25	311	0.45
(((((((Carassius_auratus_B,Carassius_gibelio_B),Cyprinus_carpio_B),Poropuntius_huangchuchieni),((Carassius_auratus_A,Carassius_gibelio_A),Cyprinus_carpio_A)),Cirrhinus_molitorella),(Ctenopharyngodon_idellus,Megalobrama_amblycephala)),Danio_rerio)	804	0.22	228	0.33
((((((Carassius_auratus_A,Carassius_gibelio_A),Cyprinus_carpio_A),Cirrhinus_molitorella),(((Carassius_auratus_B,Carassius_gibelio_B),Cyprinus_carpio_B),Poropuntius_huangchuchieni)),(Ctenopharyngodon_idellus,Megalobrama_amblycephala)),Danio_rerio)	473	0.13	91	0.13
((((((Carassius_auratus_A,Carassius_gibelio_A),Cyprinus_carpio_A),((Carassius_auratus_B,Carassius_gibelio_B),(Cyprinus_carpio_B,Poropuntius_huangchuchieni))),Cirrhinus_molitorella),(Ctenopharyngodon_idellus,Megalobrama_amblycephala)),Danio_rerio)	61	0.02	7	0.01
(((((((Carassius_auratus_A,Carassius_gibelio_A),Cyprinus_carpio_A),((Carassius_auratus_B,Carassius_gibelio_B),Cyprinus_carpio_B)),Poropuntius_huangchuchieni),Cirrhinus_molitorella),(Ctenopharyngodon_idellus,Megalobrama_amblycephala)),Danio_rerio)	60	0.02	5	0.01
(((((((Carassius_auratus_B,Carassius_gibelio_B),Poropuntius_huangchuchieni),Cyprinus_carpio_B),((Carassius_auratus_A,Carassius_gibelio_A),Cyprinus_carpio_A)),Cirrhinus_molitorella),(Ctenopharyngodon_idellus,Megalobrama_amblycephala)),Danio_rerio)	45	0.01	1	0
((((((Carassius_auratus_B,Carassius_gibelio_B),Cyprinus_carpio_B),(Cirrhinus_molitorella,Poropuntius_huangchuchieni)),((Carassius_auratus_A,Carassius_gibelio_A),Cyprinus_carpio_A)),(Ctenopharyngodon_idellus,Megalobrama_amblycephala)),Danio_rerio)	43	0.01	3	0

((((((Carassius_auratus_A,Carassius_gibelio_A),Cyprinus_carpio_A),(Ctenopharyngodon_idellus,Megalobrama_amblycephala)),(((Carassius_auratus_B,Carassius_gibelio_B),Cyprinus_carpio_B),Poropuntius_huangchuchieni)),Cirrhinus_molitorella),Danio_rerio)	38	0.01	1	0
(((((((Carassius_auratus_A,Carassius_gibelio_A),(Carassius_auratus_B,Carassius_gibelio_B)),(Cyprinus_carpio_A,Cyprinus_carpio_B)),Poropuntius_huangchuchieni),Cirrhinus_molitorella),(Ctenopharyngodon_idellus,Megalobrama_amblycephala)),Danio_rerio)	35	0.01	8	0.01
(((((((Carassius_auratus_B,Carassius_gibelio_B),Cyprinus_carpio_B),Poropuntius_huangchuchieni),((Carassius_auratus_A,Carassius_gibelio_A),Cyprinus_carpio_A)),(Ctenopharyngodon_idellus,Megalobrama_amblycephala)),Cirrhinus_molitorella),Danio_rerio)	33	0.01	1	0

Supplementary Table 15 | The shared lost genes of *C. auratus* and *C. gibelio*

**NCBI Gene ID of common crap (*Cyprinus carpio*)**	**Gene Name**	**Product name from NCBI annotation**
rna-XM_019086536.2	*lyl1*	protein lyl-1-like
rna-XM_019113279.2	*or52n2*	olfactory receptor 52N2-like
rna-XM_019119913.2	*nanos3*	nanos homolog 3-like
rna-XM_019120192.2	*pnpla8*	calcium-independent phospholipase A2-gamma isoform X1
rna-XM_042711525.1	*oacyl*	O-acyltransferase like protein-like
rna-XM_042716273.1	*npy2r*	neuropeptide Y receptor type 2-like
rna-XM_042717804.1	*bambi*	BMP and activin membrane-bound inhibitor homolog
rna-XM_042726689.1	*chrna4*	neuronal acetylcholine receptor subunit alpha-4
rna-XM_042726882.1	*acy3*	N-acyl-aromatic-L-amino acid amidohydrolase (carboxylate-forming) A
rna-XM_042734748.1	*aldoa*	fructose-bisphosphate aldolase A-like
rna-XM_042742332.1	*mdh1b*	putative malate dehydrogenase 1B
rna-XM_042751130.1	*b4galnt1a*	beta-1,4-N-acetyl-galactosaminyl transferase 1a
rna-XM_042759302.1	*LOC122145528*	uncharacterized protein LOC122145528
rna-XM_042763214.1	*mbnl2*	muscleblind-like protein 2 isoform X1
rna-XM_042764533.1	*cldn15la*	claudin 15-like a
rna-XM_042765938.1	*cdk2*	cyclin-dependent kinase 2-like [Source:NCBI gene;Acc:109084129]
rna-XM_042774944.1	*gps2*	G protein pathway suppressor 2-like
rna-XM_042775404.1	*c1qtnf4*	complement C1q tumor necrosis factor-related protein 4-like

Supplementary Table 16 | Illumina re-sequenced reads of *C. gibelio* and *C. auratus* datasets

Species	ID in main text	Raw reads			Qualified reads		
		Number of reads	Number of sequenced bases	Mean length of reads	Number of reads	Number of sequenced bases	Mean length of reads
C. gibelio	*Cg*-F1	1,016,073,664	101,607,366,400	100	1,016,073,664	101,392,547,958	99
	Cg-H1	755,025,492	113,253,823,800	150	752,682,488	111,966,130,588	149
	Cg-H2	695,144,242	104,271,636,300	150	695,144,242	103,809,817,174	149
	Cg-A1	1,737,779,942	260,666,991,300	150	1,713,848,766	254,054,999,865	148
	Cg-A2	399,556,898	59,933,534,700	150	398,486,560	59,500,973,396	149
	Cg-A3	400,644,518	60,096,677,700	150	399,756,808	59,755,753,954	149
C. auratus	*Ca*-01	1,679,904,174	251,985,626,100	150	1,679,904,174	250,850,570,624	149
	Ca-02	379,557,048	94,889,262,000	250	375,034,648	92,606,471,254	246

Supplementary Table 17 | Download data sources from NCBI

ID	Sources
C. auratus-01	This study
C. auratus-02	SRR10599079
C. auratus-03	DRR172177
C. auratus-04	DRR172189
C. auratus-05	DRR172191
C. auratus-06	DRR172193
C. auratus-07	DRR172204
C. auratus-08	DRR172206
C. auratus-09	DRR172215
C. auratus-10	DRR172218
Cyprinus carpio	ERR5285585

Supplementary Table 18 | Statistics of variation information

Chromosome ID	length	Variant number	Percent	SNP number	Percent_1	INS number	Percent_2	DEL number	Percent_3
chr1A	35,386,190	1,622,216	4.58	1,132,604	3.20	96,830	0.27	101,609	0.29
chr1B	36,562,877	1,623,602	4.44	1,136,380	3.11	98,537	0.27	101,369	0.28
chr2A	29,847,804	1,457,572	4.88	1,020,370	3.42	85,032	0.28	92,160	0.31
chr2B	30,271,480	1,406,226	4.65	982,043	3.24	84,317	0.28	88,848	0.29
chr3A	33,475,191	1,646,434	4.92	1,150,241	3.44	90,566	0.27	99,825	0.30
chr3B	50,118,171	2,252,822	4.50	1,600,538	3.19	116,806	0.23	131,168	0.26
chr4A	40,215,424	1,698,344	4.22	1,201,679	2.99	86,790	0.22	99,750	0.25
chr4B	28,104,874	1,397,191	4.97	976,682	3.48	78,985	0.28	86,398	0.31
chr5A	38,253,659	1,839,013	4.81	1,303,696	3.41	108,200	0.28	117,794	0.31
chr5B	36,161,242	1,714,833	4.74	1,202,629	3.33	104,175	0.29	112,148	0.31
chr6A	30,939,686	1,435,568	4.64	1,002,233	3.24	86,596	0.28	92,464	0.30
chr6B	29,573,676	1,357,360	4.59	951,698	3.22	84,260	0.28	88,632	0.30
chr7A	41,370,417	2,204,119	5.33	1,535,522	3.71	121,750	0.29	133,671	0.32
chr7B	42,068,215	1,854,637	4.41	1,316,268	3.13	112,426	0.27	117,103	0.28
chr8A	27,700,533	1,432,765	5.17	993,927	3.59	82,816	0.30	89,583	0.32
chr8B	30,422,264	1,345,503	4.42	943,636	3.10	81,483	0.27	87,884	0.29
chr9A	30,268,488	1,438,572	4.75	1,007,547	3.33	84,410	0.28	91,316	0.30
chr9B	35,413,027	1,476,678	4.17	1,036,693	2.93	87,925	0.25	93,861	0.27
chr10A	22,862,535	1,090,177	4.77	765,184	3.35	63,033	0.28	69,836	0.31
chr10B	23,375,766	1,122,578	4.80	774,306	3.31	66,253	0.28	71,231	0.30
chr11A	23,395,214	1,159,279	4.96	805,481	3.44	68,366	0.29	73,465	0.31
chr11B	24,479,626	1,205,789	4.93	837,044	3.42	72,574	0.30	76,894	0.31
chr12A	24,042,631	1,188,124	4.94	828,073	3.44	70,618	0.29	74,902	0.31
chr12B	23,631,608	1,095,395	4.64	762,670	3.23	67,892	0.29	70,706	0.30

chr13A	31,450,662	1,360,244	4.33	960,383	3.05	79,166	0.25	86,947	0.28
chr13B	29,417,698	1,417,146	4.82	992,670	3.37	84,378	0.29	91,856	0.31
chr14A	26,914,525	1,359,449	5.05	963,193	3.58	77,711	0.29	84,711	0.31
chr14B	28,775,669	1,291,037	4.49	909,438	3.16	78,038	0.27	80,743	0.28
chr15A	24,531,501	1,256,182	5.12	875,895	3.57	70,935	0.29	78,287	0.32
chr15B	26,664,337	1,208,261	4.53	845,151	3.17	72,443	0.27	78,485	0.29
chr16A	28,399,711	1,292,770	4.55	906,420	3.19	76,749	0.27	83,719	0.29
chr16B	30,504,434	1,358,250	4.45	950,047	3.11	83,424	0.27	87,672	0.29
chr17A	26,344,876	1,348,402	5.12	947,957	3.60	78,012	0.30	84,607	0.32
chr17B	27,353,385	1,244,834	4.55	879,530	3.22	74,387	0.27	80,271	0.29
chr18A	30,088,908	1,325,736	4.41	935,200	3.11	77,281	0.26	83,419	0.28
chr18B	28,619,747	1,397,686	4.88	978,331	3.42	82,688	0.29	88,049	0.31
chr19A	27,243,412	1,301,245	4.78	912,503	3.35	77,340	0.28	83,235	0.31
chr19B	29,195,226	1,450,777	4.97	1,010,359	3.46	85,450	0.29	90,493	0.31
chr20A	26,118,538	1,211,621	4.64	845,242	3.24	73,705	0.28	79,389	0.30
chr20B	27,371,858	1,297,130	4.74	912,059	3.33	78,136	0.29	84,035	0.31
chr21A	22,963,375	1,108,807	4.83	770,227	3.35	67,932	0.30	71,302	0.31
chr21B	26,431,221	1,262,684	4.78	881,149	3.33	74,654	0.28	80,311	0.30
chr22B	45,457,829	2,220,611	4.88	1,525,694	3.36	102,396	0.23	119,140	0.26
chr22A	22,536,000	1,160,907	5.15	808,464	3.59	60,075	0.27	66,097	0.29
chr23A	24,885,537	1,194,042	4.80	829,821	3.33	71,118	0.29	77,223	0.31
chr23B	26,272,494	1,203,483	4.58	841,504	3.20	72,682	0.28	75,958	0.29
chr24A	23,430,752	1,163,358	4.97	805,257	3.44	68,282	0.29	73,192	0.31
chr24B	23,634,917	1,174,852	4.97	811,213	3.43	69,856	0.30	74,899	0.32
chr25A	18,940,634	936,252	4.94	651,661	3.44	53,185	0.28	58,066	0.31
chr25B	24,967,067	1,179,742	4.73	826,514	3.31	64,626	0.26	71,918	0.29
Total	1,476,454,911	69,790,305	4.73	48,843,026	3.31	4,055,289	0.27	4,376,641	0.30

The table listed the variant number of different types of mutations in each chromosome. The variants were called using *C. auratus* as reference genome and the resequencing data of one *C. carpio*, 10 *C. auratus* and six *C. gibelio* individuals.

Supplementary Table 19 | List of genes with more copies in *C. gibelio* compared to *C. auratus*

Gene name	Gene number		Gene distribution	Function
	C. gibelio	*C. auratus*		
h2af1al	13	3	dispersed distribution	Core component of nucleosome.
lem4	10	3	tandem duplication	Involves in mitotic nuclear envelope reassembly by promoting dephosphorylation of BAF/BANF1 during mitotic exit.
incenp	7	2	dispersed distribution	Component of the chromosomal passenger complex (CPC), a complex that acts as a key regulator of mitosis.
rhoa	7	4	dispersed distribution	Mainly associates with cytoskeleton organization.
fbxo5	6	2	tandem duplication	Regulator of APC activity during mitotic and meiotic cell cycle.
wdr26a	5	2	dispersed distribution	G-beta-like protein involved in cell signal transduction.
tcl19l	5	2	dispersed distribution	Potential trans-activating factor that could play an important role in the transcription of genes required for the later stages of cell cycle progression.
lap2	5	2	dispersed distribution	May be involved in the structural organization of the nucleus and in the post-mitotic nuclear assembly.
c1orf159	5	2	dispersed distribution	Unknown
ccna2	4	2	dispersed distribution	Cyclin which controls both the G1/S and the G2/M transition phases of the cell cycle.
faap24	3	1	dispersed distribution	Plays a role in DNA repair through recruitment of the FA core complex to damaged DNA.
bmb	3	2	dispersed distribution	Required for nuclear membrane fusion during karyogamy.
nusap1	3	2	dispersed distribution	Microtubule-associated protein with the capacity to bundle and stabilize microtubules

Supplementary Table 20 | Clean re-sequencing data statistics of 11 individuals in a gynogenetic *C. gibelio* clonal line

Sample	GC_rate (%)	Q20_rate (%)	Q30_rate (%)	Reads (Mb)	Bases (Gb)	Clean_data/Raw_data (%)
G4-1	36.93	96.34	88.76	642.44	96.37	97.81
G4-2	36.69	95.46	86.57	461.22	69.18	96.46
G4-3	37.93	96.25	88.56	538.88	80.83	96.95
G4-4	37.99	96.54	89.31	516.60	77.49	96.63
G4-5	37.23	96.38	88.94	652.96	97.94	96.55
G4-6	37.40	97.11	90.74	630.18	94.53	95.90
G4-7	37.46	96.09	88.14	500.93	75.14	97.11
G4-8	36.91	95.57	86.74	425.56	63.83	96.64
G4-9	37.65	96.06	88.05	539.57	80.94	96.91
G4-10	37.04	95.99	87.90	491.91	73.79	97.02
G4-11	37.35	95.64	87.11	522.64	78.40	95.68

Supplementary Table 21 | Summary of LOH in a gynogenetic *C. gibelio* clonal line

Generation	All_SNP	Sites/generation	Rate	Reads depth	Region/generation	Totol_length/generation	length/genome_size
4	9780732	2283	2.33E-04	51.1	51.25	883322	5.88E-04
4	9780732	122.25	1.25E-05	39.61	57.25	190168	1.27E-04
4	9780732	6296.75	6.44E-04	49.67	31.25	2118598	1.41E-03
4	9780732	151.25	1.55E-05	42.7	55.5	156862	1.04E-04
4	9780732	6275.75	6.42E-04	50.9	23.75	2076522	1.38E-03
4	9780732	138.25	1.41E-05	37.3	65	272699	1.82E-04
4	9780732	181.75	1.86E-05	42.01	57	187781	1.25E-04
4	9780732	188.5	1.93E-05	34.74	62.5	195905	1.30E-04
4	9780732	179	1.83E-05	51.87	54.25	186644	1.24E-04
4	9780732	149.75	1.53E-05	35.18	68.25	242594	1.61E-04
4	9780732	95.25	9.74E-06	39.4	25.5	101910	6.78E-05
Average			1.49E-04				

Supplementary Table 22 | Summary of gene conversion in a gynogenetic *C. gibelio* clonal line

Generation	All_SNP	Sites/generation	Rate	Reads depth	Region/generation	Totol_length/generation	Length/genome_size
4	9780732	2191.25	2.24E-04	51.83	19.5	702725	0.000625
4	9780732	37.5	3.83E-06	52.65	23.5	11632	0.000213
4	9780732	6266.5	6.41E-04	49.76	14	2066412	0.00151
4	9780732	94.75	9.69E-06	49.39	27.5	42780	0.000239
4	9780732	6262.75	6.40E-04	50.94	12.5	2066140	0.0014
4	9780732	36	3.68E-06	48.93	22.25	15278	0.000722
4	9780732	101.5	1.04E-05	48.82	26.5	63224	0.000265
4	9780732	101.5	1.04E-05	39.46	27	63044	0.000401
4	9780732	102	1.04E-05	60.11	27	63263	0.000256
4	9780732	36	3.68E-06	47.7	22	11534	0.000445
4	9780732	23.75	2.43E-06	52.33	13	25330	0.000114
Average			1.42E-04				

Supplementary Table 23. Redundancy comparison of different versions of *C. auratus* assembly.

Genome assembly version	Mapping rate of shot reads	Mapping rate of transcriptomic reads	Reads used for purge	Genome length before purge (bp)	Genome length after purge (bp)
This study	99.85%	85.56%~89.91%	154Gb short reads	1,602,881,788	1,522,436,151
SA2019 (GCF_003368295.1)	99.68%	No reads available	252Gb long reads	1,820,635,050	1,488,718,052
PNAS (GCA_014332655.1)	98.90%	No reads available	140Gb long reads	1,739,639,290	1,528,230,182
SA2020(GWHAAIA00000000)	99.29%	No reads available	154Gb short reads	1,639,403,616	1,524,360,420
BMC (GCA_013115835.1)	98.23%	62.08%~90.40%	162Gb short reads	2,197,795,230	1,406,905,302
