# Supplementary material for: Comparative genome anatomy reveals evolutionary insights into a unique amphitriploid fish
Source: Nat Ecol Evol. 2022 Jul 11;6(9):1354–66. doi: 10.1038/s41559-022-01813-z (PMC9439954; doi:10.1038/s41559-022-01813-z)
Supplement: Supplementary file 2 — Reporting Summary [file 41559_2022_1813_MOESM2_ESM.pdf]

nature portfolio

Corresponding author(s): Jian-Fang Gui

Last updated by author(s): May 19, 2022

Reporting Summary

Nature Portfolio wishes to improve the reproducibility of the work that we publish. This form provides structure for consistency and transparency in reporting. For further information on Nature Portfolio policies, see our Editorial Policies and the Editorial Policy Checklist.

Statistics

For all statistical analyses, confirm that the following items are present in the figure legend, table legend, main text, or Methods section.

n/a	Confirmed	
☐	☒	The exact sample size (*n*) for each experimental group/condition, given as a discrete number and unit of measurement
☐	☒	A statement on whether measurements were taken from distinct samples or whether the same sample was measured repeatedly
☐	☒	The statistical test(s) used AND whether they are one- or two-sided<br>*Only common tests should be described solely by name; describe more complex techniques in the Methods section.*
☐	☒	A description of all covariates tested
☐	☒	A description of any assumptions or corrections, such as tests of normality and adjustment for multiple comparisons
☐	☒	A full description of the statistical parameters including central tendency (e.g. means) or other basic estimates (e.g. regression coefficient) AND variation (e.g. standard deviation) or associated estimates of uncertainty (e.g. confidence intervals)
☐	☒	For null hypothesis testing, the test statistic (e.g. *F*, *t*, *r*) with confidence intervals, effect sizes, degrees of freedom and *P* value noted<br>*Give P values as exact values whenever suitable.*
☐	☒	For Bayesian analysis, information on the choice of priors and Markov chain Monte Carlo settings
☐	☒	For hierarchical and complex designs, identification of the appropriate level for tests and full reporting of outcomes
☐	☒	Estimates of effect sizes (e.g. Cohen's *d*, Pearson's *r*), indicating how they were calculated

Our web collection on statistics for biologists contains articles on many of the points above.

Software and code

Policy information about availability of computer code

Data collection	Software was only used for data analyses.
Data analysis	All the softwares used for analysis have been described in the Online Methods as well as Supplementary Methods. All software used in this study included: NextDenovo v2.3.1, Nextpolish v1.3.1, Juicer v 1.6, 3D-DNA v180922, Juicebox Assembly Tools 1.9.8, Purge_dups v1.0.1, BWA v0.7.17-r1198-dirty, SAMtools v1.12, BEDTools, BUSCO v5.2.2, LTR FINDER v1.0.5, TRF v4.07b, RepeatMasker v4.0.5, RepeatProteinMask v1.36, RepeatModeler v1.0.8, AUGUSTUS v3.2.1, BLAST 2.10.1, GeneWise v2.2.0, HISAT2 v2.1.0, StringTie v2.1.4, Cufflinks v5.5.0, InterProscan-5.16-55.0, SOAPdenovoso2-r244, MCScan v0.8, FreeBayes v0.9.10-3-g47a713e, MUSCLE v3.8.42, pal2nal.v14, Gblocks v0.91b, ASTRAL v5.7.1, DensiTree, PAML v4.9h, ASTRAL v5.7.1, KaKs_Calculator2.0, Phybase v.1.5, RAxML-8.2.12, SnpEff v4.3t, MEGA v7.0.26, FigTree v1.4.4, mafft v7.471.

For manuscripts utilizing custom algorithms or software that are central to the research but not yet described in published literature, software must be made available to editors and reviewers. We strongly encourage code deposition in a community repository (e.g. GitHub). See the Nature Portfolio guidelines for submitting code & software for further information.

Data

Policy information about availability of data

All manuscripts must include a data availability statement. This statement should provide the following information, where applicable:

- Accession codes, unique identifiers, or web links for publicly available datasets
- A description of any restrictions on data availability
- For clinical datasets or third party data, please ensure that the statement adheres to our policy

The whole genome assembly and the raw resequencing data of C. gibelio are deposited into GenBank under BioProject ID PRJNA546443. The whole genome

assembly and the raw resequencing data of C. auratus are deposited into GenBank under BioProject ID PRJNA546444. The transcriptome data of C. gibelio and C. auratus are available in the GenBank (PRJNA836313, PRJNA834570, PRJNA833164, PRJNA837728, PRJNA833750, and PRJNA833167). The gene alignments and trees of specific lost and expanded genes are available at figshare database (https://doi.org/10.6084/m9.figshare.19674843.v1).

Field-specific reporting

Please select the one below that is the best fit for your research. If you are not sure, read the appropriate sections before making your selection.

☐ Life sciences ☐ Behavioural & social sciences ☒ Ecological, evolutionary & environmental sciences

For a reference copy of the document with all sections, see nature.com/documents/nr-reporting-summary-flat.pdf

Ecological, evolutionary & environmental sciences study design

All studies must disclose on these points even when the disclosure is negative.

Study description	Triploids are generally considered as an evolutionary "dead-end" because of two major biological difficulties for meiotic pairing and segregation of three homologous chromosomes and for purging deleterious mutations due to the lack of meiotic recombination. However, triploids are found in some polyploid complex (coexistence of diploid, triploid, tetraploid, or hexaploid) species, which usually have overcome the reproductive obstacles via unisexual production. However, the evolutionary mechanisms underpinning the gynogenetic system have largely remained unknown. Here, we used Carassius complex of cyprinid teleost to illustrate this enigma, which contains sexual tetraploid C. auratus and unisexual hexaploid C. gibelio.<br>Firstly, we sequenced the genomes of these two species, and managed to assemble their haplotypes that contain two subgenomes (AB) to the chromosome level. Sequencing coverage analysis reveals that C. gibelio is an amphitriploid (AAABBB) with two triploid sets of chromosomes; each set is derived from a different ancestor. And the evolution process of ancient polyploidy has been characterized in the Carassius complex. Resequencing data from different strains of C. gibelio show that unisexual reproduction has been maintained for over 0.82 million years. Secondly, comparative genomic results reveal significant genomic changes specific to unisexual reproduction success, in which many meiotic cell cycle-related genes and an oocyte-specific histone variant gene family are largely expanded in the amphitriploid C. gibelio. This study provides the first genomic evidence for the novel hypothesis that a genomic assemblage and an alternative reproductive module might be required for the formation of a functioning asexual/unisexual genome as suggested by Hojsgaard and Schartl (2021). Thirdly, cytological assays indicate that C. gibelio produces unreduced oocytes by an alternative ameiotic pathway; however, sporadic homologous recombination and a high rate of gene conversion also exist in C. gibelio. These genomic changes might have facilitated purging deleterious mutations and maintaining genome stability in this unisexual amphitriploid fish. Our findings shed novel lights onto the evolutionary mechanisms underpinning the reproduction success in unisexual polyploid vertebrates.
Research sample	A female adult individual from strain F of Carassius gibelio and a female adult individual of Carassius auratus were used for de nova genome assembly. Six female C. gibelio individuals from three strains in total were used to study the origin of this unisexual species, including three female individuals of strain A+, two female individuals of strain H, and one female individual of strain F. Eleven female individuals from the offspring of the fourth generation belonging to a gynogenetic line of strain F of C. gibelio were used to perform analysis of gene conversion.
Sampling strategy	Blood cells from all samples were used for genome sequencing.
Data collection	All data were collected in the author's laboratory or downloaded from NCBI Genbank and the literature described in the manuscript.
Timing and spatial scale	from Mar, 2018 to Nov 2020
Data exclusions	No data collected were excluded from analyses.
Reproducibility	All attempts to repeat the experiment were successful.
Randomization	Samples were randomly selected from healthy adults.
Blinding	No blinding was required.

Did the study involve field work? ☐ Yes ☒ No

Reporting for specific materials, systems and methods

We require information from authors about some types of materials, experimental systems and methods used in many studies. Here, indicate whether each material, system or method listed is relevant to your study. If you are not sure if a list item applies to your research, read the appropriate section before selecting a response.

Materials & experimental systems

n/a	Involved in the study
☒	☐ Antibodies
☒	☐ Eukaryotic cell lines
☒	☐ Palaeontology and archaeology
☐	☒ Animals and other organisms
☒	☐ Human research participants
☒	☐ Clinical data
☒	☐ Dual use research of concern

Methods

n/a	Involved in the study
☒	☐ ChIP-seq
☒	☐ Flow cytometry
☒	☐ MRI-based neuroimaging

Animals and other organisms

Policy information about studies involving animals; ARRIVE guidelines recommended for reporting animal research

Laboratory animals	A female adult individual from strain F of Carassius gibelio (two years old) and a female adult individual of Carassius auratus (two years old) were used for de nova genome assembly. Six female C. gibelio individuals from three strains in total were used to study the origin of this unisexual species, including three female individuals of strain A+(two years old), two female individuals of strain H (two years old), and one female individual of strain F (two years old). Eleven female individuals from the offspring of the fourth generation belonging to a gynogenetic line of strain F of C. gibelio (two years old) were used to perform analysis of gene conversion.
Wild animals	Our study did not sample wild animals.
Field-collected samples	Our study did not include field-collected samples.
Ethics oversight	No ethical approval was required.

Note that full information on the approval of the study protocol must also be provided in the manuscript.
